# Inflammation-induced PELP1 expression promotes tumorigenesis by activating GM-CSF paracrine secretion in the tumor microenvironment

**DOI:** 10.1016/j.jbc.2021.101406

**Published:** 2021-11-11

**Authors:** Veena Kumari Vuttaradhi, Inemai Ezhil, Divya Ramani, Rahul Kanumuri, Swetha Raghavan, Vaishnavi Balasubramanian, Roshni Saravanan, Archana Kanakarajan, Leena Dennis Joseph, Ravi Shankar Pitani, Sandhya Sundaram, Anita Sjolander, Ganesh Venkatraman, Suresh Kumar Rayala

**Affiliations:** 1 Molecular Oncology Laboratory, Department of Biotechnology, Indian Institute of Technology Madras, Chennai, Tamil Nadu, India; 2 Department of Human Genetics, Sri Ramachandra Faculty of Biomedical Sciences & Technology, Sri Ramachandra Institute of Higher Education and Research, Chennai, Tamil Nadu, India; 3 Department of Pathology, Sri Ramachandra Medical College, Sri Ramachandra Institute of Higher Education and Research, Chennai, Tamil Nadu, India; 4 Department of Community Medicine, Sri Ramachandra Medical College, Sri Ramachandra Institute of Higher Education and Research, Chennai, Tamil Nadu, India; 5 Cell Pathology, Department of Translational Medicine, Clinical Research Centre, Skåne University Hospital, Lund University, Malmö, Sweden

**Keywords:** chronic inflammation, c-Rel, GM-CSF, paracrine regulation, PELP1, cDNA, complementary DNA, ChIP, chromatin immunoprecipitation, CM, conditioned media, DMEM, Dulbecco's modified Eagle's medium, DSS, dextran sulfate sodium, FACS, fluorescent-activated cell sorting, FBS, fetal bovine serum, GM-CSF, granulocyte–macrophage colony-stimulating factor, HRP, horseradish peroxidase, IFN-γ, interferon gamma, IHC, immunohistochemistry, IL, interleukin, JAK, Janus kinase, KD, knockdown, LPS, lipopolysaccharide, MAPK, mitogen-activated protein kinase, NSAID, nonsteroidal anti-inflammatory drug, PELP1, proline, glutamic acid, and leucine-rich protein 1, Ptx, pentoxifylline, qRT, quantitative RT, STAT3, signal transducer and activator of transcription 3, TNF-α, tumor necrosis factor alpha

## Abstract

The inflammatory tumor microenvironment has been implicated as a major player fueling tumor progression and an enabling characteristic of cancer, proline, glutamic acid, and leucine-rich protein 1 (PELP1) is a novel nuclear receptor coregulator that signals across diverse signaling networks, and its expression is altered in several cancers. However, investigations to find the role of PELP1 in inflammation-driven oncogenesis are limited. Molecular studies here, utilizing macrophage cell lines and animal models upon stimulation with lipopolysaccharide (LPS) or necrotic cells, showed that PELP1 is an inflammation-inducible gene. Studies on the PELP1 promoter and its mutant identified potential binding of c-Rel, an NF-κB transcription factor subunit, to PELP1 promoter upon LPS stimulation in macrophages. Recruitment of c-Rel onto the PELP1 promoter was validated by chromatin immunoprecipitation, further confirming LPS mediated PELP1 expression through c-Rel–specific transcriptional regulation. Macrophages that overexpress PELP1 induces granulocyte–macrophage colony-stimulating factor secretion, which mediates cancer progression in a paracrine manner. Results from preclinical studies with normal–inflammatory–tumor progression models demonstrated a progressive increase in the PELP1 expression, supporting this link between inflammation and cancer. In addition, animal studies demonstrated the connection of PELP1 in inflammation-directed cancer progression. Taken together, our findings provide the first report on c-Rel–specific transcriptional regulation of PELP1 in inflammation and possible granulocyte–macrophage colony-stimulating factor–mediated transformation potential of activated macrophages on epithelial cells in the inflammatory tumor microenvironment, reiterating the link between PELP1 and inflammation-induced oncogenesis. Understanding the regulatory mechanisms of PELP1 may help in designing better therapeutics to cure various inflammation-associated malignancies.

The connection between chronic inflammation and cancer was first proposed by Rudolf Virchow in 1863, who elucidated that tumors emerge at the sites of inflammation with the evidence of infiltrated immune cells in tumor samples (1). Tumor-promoting inflammation has widely acquired prominence now as a cancer hallmark contributing to tumor progression by stimulating proliferation, cancer survival, angiogenesis, tumor metastasis, and immune circumvention (2). Chronic inflammation fosters consistent acquisition of mutations and epigenetic variations (3) that induce genetic alterations in cells that trigger transcriptional activity of various proteins like NF-κB (4, 5), signal transducer and activator of transcription 3 (STAT3), and hypoxia-inducible factor 1α (6, 7) in tumor cells. These proteins cooperate to produce inflammatory mediators like cytokines and chemokines, which sequentially recruit and activate various immune cells (8). The interactions among the immune cells, cancer cells, and the surrounding microenvironment play a crucial role in cancer-associated inflammation (9). Macrophages are the predominant immune cells that play a pivotal role in the orchestration of inflammation-related cancer and function as prime producers of proinflammatory cytokines that affect cancer progression (9, 10).

Proline, glutamic acid, and leucine-rich protein 1 (PELP1) is a novel nuclear receptor coregulator that serves various signaling activities (11). PELP1 works as a scaffolding molecule that integrates several signaling proteins with steroid hormone receptors (12). Although PELP1 was initially identified as an estrogen receptor coregulator (13), further studies have established PELP1 as a vital regulator for numerous transcription factors (14); it is also associated with chromatin remodeling (15), RNA processing (16), and ribosome biogenesis (17). Emerging evidences suggest the potential of PELP1 as an important driver of proliferation in cancer (18). PELP1 is a proto-oncogene, which interacts and regulates the functional activity of various proto-oncogenes, like STAT3, c-Src, epidermal growth factor receptor, and so on (19, 20). PELP1 displays its function as a key coregulatory protein offering tumor cells with a specific growth and survival preference by modulating proliferation, migration, invasion, metastasis, and endocrine resistance (21, 22). PELP1 is involved in the progression of different cancers, including endometrial, salivary, ovarian, pancreas, prostate, colon, lung, and also mainly breast cancers (23, 24). A recent report suggests that cytoplasmic PELP1 induces macrophage activation, which promotes epithelial cell migration in a paracrine manner through inflammatory cytokines and chemokines *via* NF-κB, inducing protumorigenic signaling associated with cancer initiation (25). A plenitude of data describes the role of PELP1 in tumor development, but limited investigation has been carried out on unraveling its role in inflammation-driven oncogenesis.

The aim of the current report is to explore the molecular mechanism of PELP1 induction during inflammation and its role in subsequent progression to cancer. Understanding the molecular mechanism of transcriptional regulation of PELP1 in inflammation can throw light on the evolution of new therapeutics to alleviate numerous malignancies associated with chronic inflammation. To achieve this, we have utilized both human as well as mouse macrophage cell lines and identified c-Rel, a NF-κB transcription factor, as the molecule responsible for PELP1 induction during inflammation. Furthermore, we distinctly established granulocyte–macrophage colony-stimulating factor (GM-CSF) as a novel and promising downstream target of PELP1 through quantitative RT (qRT)–PCR expression profiling array. We also provided critical evidence of the involvement of GM-CSF in cancer transformation through various *in vitro* cellular transformation assays. To further support the *in vitro* findings, we demonstrated expression of PELP1 in clinical samples comprising of normal–inflammatory–cancer progression disease spectra. In addition, we utilized an *in vivo* animal model system to augment the clinical significance of PELP1 in inflammation and cancer progression.

## Results

### PELP1 is an inflammatory responsive gene

**Figure 1:**
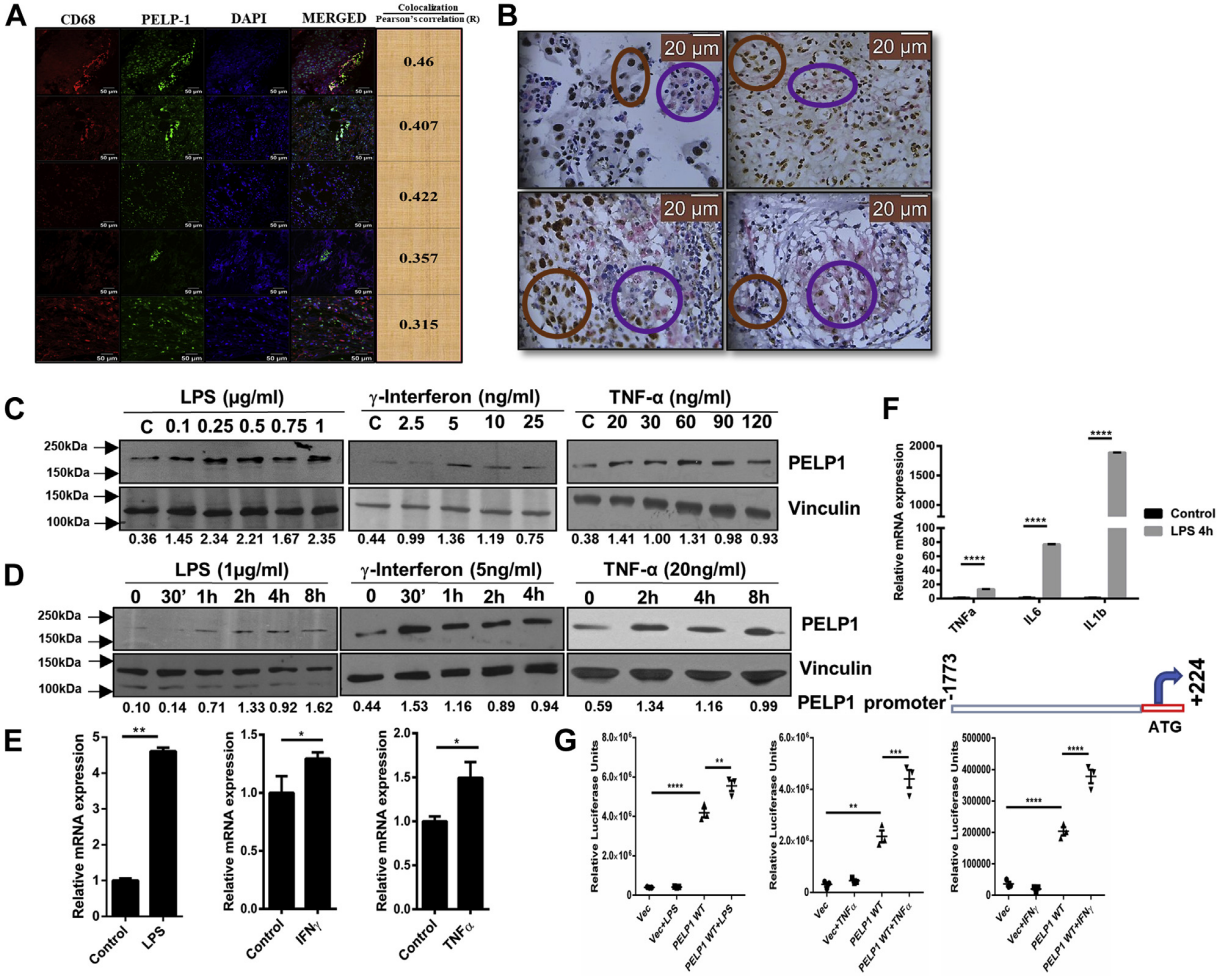
**PELP1 is an inflammatory responsive gene.***A* and *B*, representative double immunofluorescence (IF) images and double IHC images to show the expression of PELP1 in CD-68 positive cells in inflammatory breast tumor. In double IF, PELP1 was detected with Alexa Fluor 488 (*green*) and CD68 with Alexa Fluor 546 (*red*). The merged images show the coexpressed cells in *yellow* (the mean Pearson's correlation coefficient was calculated to be 0.2481, n = 13). For double IHC, PELP1 expression was nuclear localized as seen in DAB-positive cells, and CD68 was found mostly in the cytoplasm in close vicinity to the tumor as seen in Alk positive cells. The *brown rings* in (*B*) show the PELP1-positive regions, and *magenta rings* show the CD68-positive regions. The mean coexpression percentage of cells expressing both PELP1 and CD68 was calculated to be 23.85% (n = 13). *C* and *D*, Western blot images of PELP1 from RAW 264.7 cells treated with LPS, IFN-γ, and TNF-α in a concentration-dependent and time-dependent manner with vinculin as a loading control. *E*, PELP1 mRNA expression normalized with β-actin from RAW 264.7 cells treated with LPS (1 μg/ml) for 4 h and IFN-γ (5 ng/ml) and TNF-α (20 ng/ml) for 8 h time intervals. *F*, quantitative RT–PCR analysis showing the expression of three different inflammatory cytokines (TNF-α, IL-6, and IL-1β) under LPS (1 μg/ml for 4 h) treatment in RAW 264.7 cells. The expression of β-actin was used for normalization. *G*, *top*, schematic representation of PELP1 promoter cloned upstream of luciferase gene in the multiple cloning site (MCS) of pGL3 basic vector using HindIII and XhoI restriction sites. *Bottom*, relative luciferase activity of PELP1 promoter in RAW 264.7 cells on treatment with LPS (1 μg/ml for 4 h), TNF-α (30 ng/ml for 8 h), and IFN-γ (5 ng/ml for 8 h). For all the experiments, the data are represented as mean ± SEM and ∗ (*p* ≤ 0.05), ∗∗ (*p* ≤ 0.01), ∗∗∗ (*p* ≤ 0.001), and ∗∗∗∗ (*p* < 0.0001) for presenting *p* values (unless otherwise specified). DAB, 3,3′-diaminobenzidine; IFN-γ, interferon gamma; IHC, immunohistochemistry; IL, interleukin; LPS, lipopolysacharide; PELP1, proline, glutamic acid, and leucine-rich protein 1; TNF-α, tumor necrosis factor alpha.

Macrophages represent the major components of inflammatory infiltrate of various chronic inflammation–related cancers and produce several potent tumor-inducing signals, thus displaying a significant role in cancer development. To assess the expression of PELP1 in macrophages present in tumor milieu, we analyzed the expression of CD68, a macrophage-specific marker along with PELP1 in serial sections of inflammatory breast tumor tissues (N = 13) by double immunohistochemistry (IHC) staining and double immunofluorescence. Most of the CD68-positive cells also expressed PELP1. In double immunofluorescence, PELP1 was visualized with Alexa Fluor 488 (*green*) and CD68 by Alexa Fluor 546 (*red*). Super imposing these immunostained tissue sections exhibited strong coexpression (*yellow*) patterns of both PELP1 and CD68. The coexpression analysis was performed using ImageJ software (NIH), and the mean Pearson's correlation coefficient was calculated to be 0.2481 (N = 13). In the case of double IHC, breast tumor cells demonstrated nuclear PELP1 expression (3,3′-diaminobenzidine; *brown*) and CD68 expression (Alk *magenta*) in the cytoplasm, indicating macrophages. The mean Q score for tumor cells showing PELP1 expression was found to be 101.54 (n = 13). Coexpression percentage was determined by those cells that exhibited brown nucleus (PELP1) and pink cytoplasm (CD68). The mean coexpression percentage for cells was found to be 23.85% (n = 13). Figure 1*A* is a representative panel of double immunofluorescence staining and Figure 1*B* for double IHC staining. Next, to assess the inflammatory responsiveness of PELP1, RAW 264.7, a murine macrophage cell line, was stimulated with well-known inflammatory inducers like lipopolysaccharide (LPS)/interferon gamma (IFN-γ)/tumor necrosis factor alpha (TNF-α) in a concentration- and time-dependent manner. These lysates were checked for expression of PELP1 at protein and mRNA levels by Western blot and qRT–PCR analysis, respectively. The results showed a significant induction of PELP1 at protein as well as mRNA levels with all the inflammatory cytokines tested (Fig. 1, *C*–*E*). In addition, to understand whether this induction of PELP1 was at the promoter level, we cloned the putative full-length human PELP1 promoter sequence (∼2.0 kb) into a pGL3-luciferase reporter system. RAW 264.7 cells were transfected with PELP1 promoter and stimulated with three different inflammation inducers (LPS/IFN-γ/TNF-α). Our results showed an increase in the PELP1 promoter–luciferase activity in stimulated cells, further confirming PELP1 as an inflammatory responsive gene (Fig. 1*G*).

**Figure 2:**
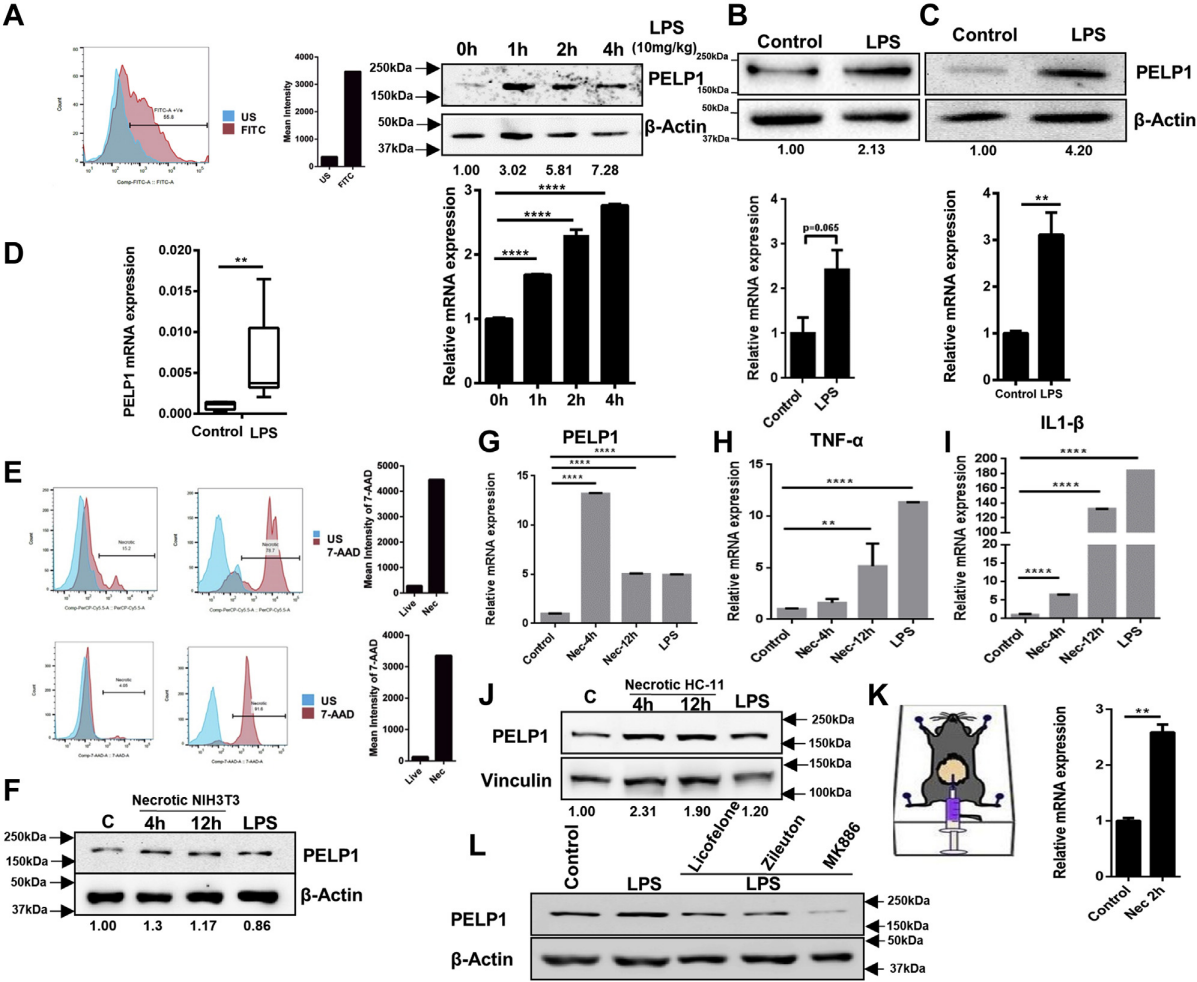
**PELP1 is an inflammatory responsive gene.***A*, *top*, FACS analysis of F4/80 (macrophage-specific marker) staining to confirm the macrophage population in the peritoneal fluid; Western blot (*middle*) and quantitative RT (qRT)–PCR (*bottom*) analysis of PELP1 in peritoneal macrophages isolated from LPS (10 mg/kg body weight) injected female BALB/c mice (6–8 weeks old) at different time intervals. *B*, Western blot (*top*) and qRT–PCR (*bottom*) analysis of PELP1 in mouse macrophage cells (MMa-bm) treated with LPS (1 μg/ml for 4 h). *C*, protein (*upper*) and mRNA (*bottom*) expression of PELP1 in a human macrophage cell line, MD-CRL9850 treated with LPS (1 μg/ml for 4 h). *D*, PELP1 mRNA expression level in total zebrafish lysate after intraperitoneal injections with LPS for 12 h at a concentration of 20 μg/fish. *E*, FACS analysis showing live and necrotic NIH3T3 cells (*top*) and live and necrotic HC-11 cells (*bottom*) by 7-AAD staining. Necrosis was induced by repeated freeze–thawing cycles (US stands for unstained). *F*, Western blot image of PELP1 from RAW 264.7 cells incubated with necrotic NIH3T3 lysate for two different periods; here, LPS served as a positive control for PELP1 induction, and β-actin was utilized as an internal loading control. *G*, qRT–PCR data of PELP1 mRNA expression levels along with positive control genes. *H* and *I*, TNF-α and IL-1β from RAW 264.7 cells incubated with necrotic NIH3T3 lysate for two different periods. *J*, Western blot image of PELP1 from RAW 264.7 cells incubated with necrotic HC11 lysate for two different periods and LPS as a positive control for PELP1 induction. Vinculin served as an internal loading control. *K*, qRT–PCR analysis of PELP1 in peritoneal macrophages from necrotic HC11-injected female BALB/c mice of 6 to 8 weeks old. *L*, Western blot image showing PELP1 expression in RAW 264.7 cells when pretreated with three different NSAIDs, that is, licofelone (10 μM/ml), zileuton (1 μM/ml), and MK886 (5 μg/ml) for 1 h, prior to LPS (1 μg/ml for 4 h) treatment. Actin was used as a control. For all the experiments, the data are represented as mean ± SEM and ∗ (*p* ≤ 0.05), ∗∗ (*p* ≤ 0.01), ∗∗∗ (*p* ≤ 0.001), and ∗∗∗∗ (*p* < 0.0001) for presenting *p* values (unless otherwise specified). 7-AAD, 7-aminoactinomycin D; FACS, fluorescent-activated cell sorting; IL-1β, interleukin 1β; LPS, lipopolysaccharide; NSAID, nonsteroidal anti-inflammatory drug; PELP1, proline, glutamic acid, and leucine-rich protein 1; TNF-α, tumor necrosis factor alpha.

As LPS is a classic and the most studied pathogen-associated molecule that induces inflammatory responses (26), we further studied the detailed mechanism of PELP1 induction with LPS stimulation. To ascertain the molecular changes of inflammation, we analyzed the expression of three candidate cytokine genes, *viz*, TNF-α, interleukin 6 (IL-6), and IL-1β (positive control genes of inflammatory responses) in RAW 264.7 cells upon LPS treatment (Fig. 1*F*). Next, to understand the universal relevance of PELP1 expression during inflammation, we conducted experiments in various cell lines and animal models. We collected peritoneal macrophages from BALB/c female mice stimulated with LPS and analyzed for PELP1 expression. The results showed a remarkable upregulation in PELP1 at both protein and mRNA expression levels. Here, the presence of macrophages in the peritoneal fluid was confirmed by F4/80 (mouse macrophage-specific marker) staining by fluorescent-activated cell sorting (FACS) analysis (Fig. 2*A*). Furthermore, we wanted to extend our study to other cell line models of mouse origin and used the mouse primary macrophage cells differentiated from circulating bone marrow–derived monocytes (MMa-bm). The results showed an increase in PELP1 expression when induced with LPS (Fig. 2*B*). Next, to study the PELP1 induction in human macrophage system, we used the human macrophage cell line MD-CRL9850 (Fig. 2*C*), which also showed PELP1 induction upon treatment with LPS. Furthermore, we also showed a notable increase in PELP1 mRNA expression levels on stimulation with LPS in another *in vivo* zebrafish model (Fig. 2*D*).

As inflammation is a physiological response to either infection or tissue injury (27), we decided to investigate the induction of PELP1 during tissue damage. Cell death by necrosis *in vivo* can stimulate inflammatory response known as sterile inflammation (28). To mimic sterile inflammatory conditions *in vitro*, we prepared necrotic lysates of NIH3T3 and HC11 by alternate freeze–thaw cycles and confirmed necrosis using FACS analysis of 7-aminoactinomycin D–stained necrotic and live NIH3T3 and HC11 cells (Fig. 2*E*). When these necrotic cell lysates of NIH3T3 were incubated with RAW 264.7 cells, the expression of PELP1 was increased compared with the control cells at protein (Fig. 2*F*) and mRNA levels (Fig. 2*G*). We also checked the expression of two inflammatory responsive genes, namely TNF-α and IL-1β as positive control (Fig. 2, *H* and *I*). Similarly, we observed an induction in PELP1 expression when necrotic HC11, a mouse mammary epithelial cell line, was incubated with RAW 264.7 cells (Fig. 2*J*). Furthermore, the *in vivo* experiments with peritoneal macrophages isolated from female BALB/c mice when injected with necrotic HC11 cells also showed an increase in PELP1 expression compared with the control mice (Fig. 2*K*). All these findings with LPS and necrotic lysate induced inflammation and stimulated PELP1 overexpression in various *in vivo* and *in vitro* models.

To emphasize the importance of PELP1 during inflammation, we used nonsteroidal anti-inflammatory drugs (NSAIDs), which are the commonly advised medications for treating inflammatory conditions (29, 30). When RAW 264.7 cells were pretreated with three different NSAIDs—licofelone, zileuton, and MK886 prior to LPS treatment, the cells exhibited reduction in PELP1 expression compared with untreated and LPS-treated cells (Fig. 2*L*). All these observations manifest the significance of PELP1 induction during inflammation.

### c-Rel is the specific transcription factor that regulates PELP1 expression

**Figure 3:**
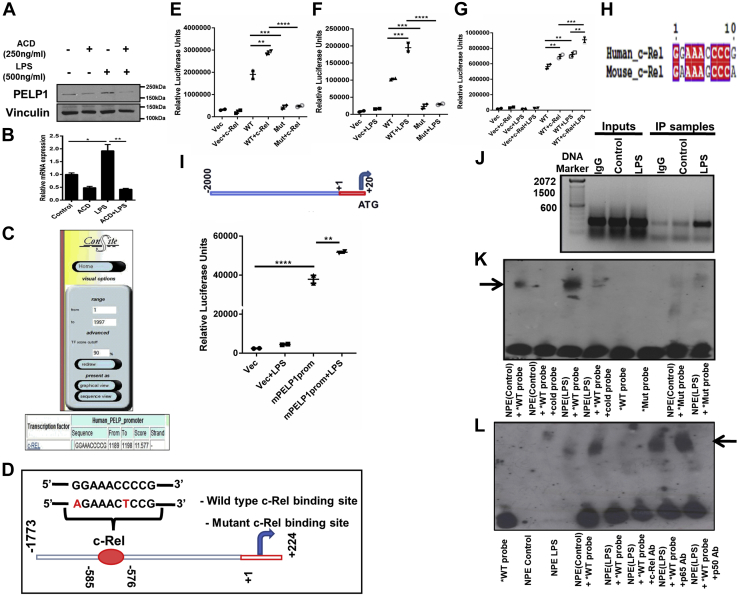
**c-Rel is the specific transcription factor regulating PELP1 expression.***A* and *B*, Western blot and quantitative RT–PCR data of PELP1 in RAW 264.7 cells on LPS (1 μg/ml) treatment when pretreated with actinomycin-D (250 ng/ml) for 4 h. *C*, sequence analysis of mouse PELP1 promoter for potential transcription factor binding sites using ConSite. *D*, schematic representation of recruitment of c-Rel onto the consensus sequence present in PELP1 promoter along with mutated binding sites, which were highlighted. *E*–*G*, relative luciferase activity of PELP1 promoter from RAW 264.7 cotransfected with either PELP1 WT or mutant (Mut) promoter and c-Rel expression plasmids along with or without LPS (500 ng/ml) treatment. *H*, the c-Rel-binding consensus sequence from human PELP1 promoter was compared with the c-Rel-binding sequence in mouse PELP1 promoter using three different multiple sequence alignment programs (MAFFT, T-COFFEE, and ESPript 3.0) that showed 80% similarity between the two c-Rel-binding sequences. *I*, schematic representation of mouse PELP1 promoter cloned upstream of luciferase gene in the multiple cloning site (MCS) of pGL3 basic vector using KpnI and NheI restriction sites and relative luciferase activity of mouse PELP1 promoter on treatment with LPS of 1 μg/ml concentration for 4 h. *J*, *in vivo* ChIP assay—an agarose gel image of PCR products obtained from immunoprecipitated chromatin of LPS-treated and untreated RAW 264.7 cells using anti c-Rel antibody and simultaneous extraction of DNA followed by PCR amplification using ChIP-specific PELP1 promoter primers (forward 5′-TTGGTGGAGGCCTTTG-3′, reverse 5′-GTTTCCGGAGGTGGTT-3′). *K*, *in vitro* EMSA assay using nuclear protein extracts (NPEs) from LPS-treated and untreated RAW 264.7 cells incubated with biotinylated WT probes either with or without nonbiotinylated WT probes (cold probes) in higher concentration to biotinylated WT probes and mutant biotinylated probes (Mut probes). *L*, super shift EMSA assay in which DNA–protein complexes were further incubated with protein-specific antibody (here, its anti-c-Rel antibody) or negative control antibodies (anti-p65 and anti-p50 antibodies) to observe either band shift or band disappearance. We observed disappearance of the specific band when the probe, and nuclear protein complexes were incubated with c-Rel-specific antibody. The *bold arrow* represents specific band of interest. ChIP, chromatin immunoprecipitation; LPS, lipopolysaccharide; PELP1, proline, glutamic acid, and leucine-rich protein 1.

To understand if PELP1 induction requires transcriptional activity, cells were treated with transcriptional inhibitor actinomycin-D; results showed that PELP1 expression was indeed through transcriptional regulation (Fig. 3, *A* and *B*). To delineate the molecular mechanism of PELP1 induction by inflammatory cytokines, we explored potential transcription factor binding sites that are present in PELP1 promoter using ConSite (Fig. 3*C*). Analysis of the PELP1 promoter sequence revealed a potential 10 bp c-Rel-binding consensus region GGAAACCCCG at 576 to 585 bp upstream of transcription start site (Fig. 3*D*). As the role of c-Rel in inflammation is well characterized (31), we speculated that c-Rel could be an ideal factor involved in the transcriptional regulation of PELP1 during inflammation. To elucidate the functional importance of this c-Rel-binding consensus sequence in the regulation of PELP1 transcriptional activity through c-Rel and LPS, we performed cotransfection studies with c-Rel and PELP1 promoter. Cotransfection of c-Rel with human PELP1 WT promoter showed an increased luciferase activity, whereas the c-Rel-binding mutant did not show any increase in the luciferase activity. In addition, PELP1 promoter with mutant c-Rel-binding site did not respond to LPS (Fig. 3, *E*–*G*). These results suggest that the functional interaction between c-Rel and its binding site GGAAACCCCG in the PELP1 promoter was required for the LPS-mediated induction of PELP1. We compared the c-Rel-binding consensus sequence from human PELP1 promoter with mouse PELP1 promoter, which showed 80% similarity between the two c-Rel-binding sequences (Fig. 3*H*). We further cloned 2 kb of mouse PELP1 promoter into pGL3 basic vector and observed an induction in luciferase activity when transfected into RAW 264.7 cells and treated with LPS (Fig. 3*I*).

**Figure 4:**
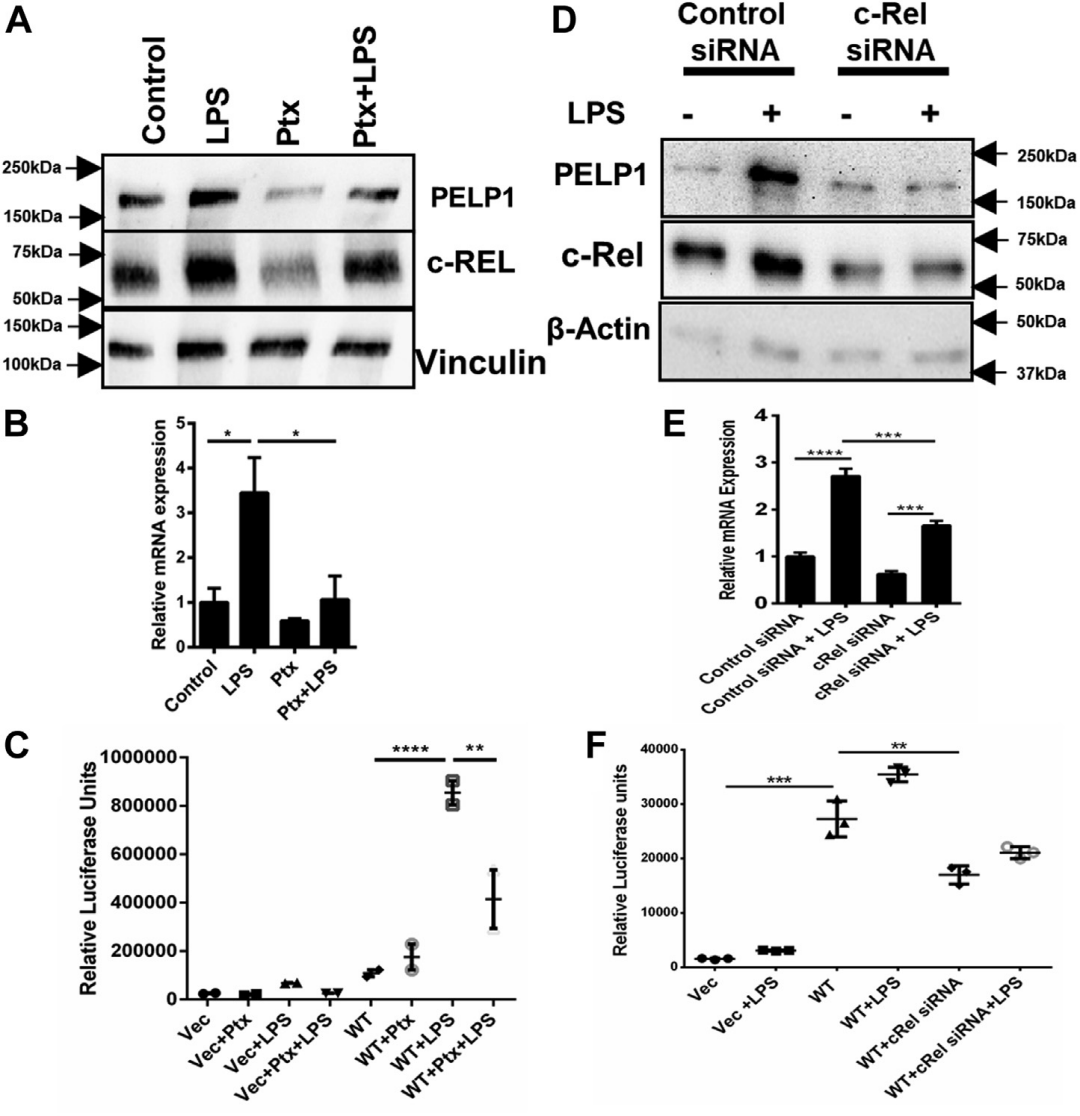
**c-Rel is the specific transcription factor regulating PELP1 expression.***A* and *B*, Western blot and quantitative RT–PCR analysis of PELP1. *C*, luciferase activity of PELP1 promoter in RAW 264.7 cells when pretreated with pentoxifylline (Ptx) for 1 h at 1 mg/ml concentration, followed by LPS (1 μg/ml) treatment for 4 h. *D* and *E*, Western blot and quantitative RT–PCR images of PELP1. *F*, luciferase activity of PELP1 promoter in RAW 264.7 cells when transfected with c-Rel siRNA at 50 pmol/well using Lipofectamine RNAiMax followed by LPS treatment of 1 μg/ml for 4 h. LPS, lipopolysaccharide; PELP1, proline, glutamic acid, and leucine-rich protein 1.

To corroborate the molecular connection between LPS and c-Rel in the modulation of transcriptional activity of PELP1, we analyzed the incorporation of c-Rel onto the PELP1 promoter comprising the c-Rel-binding sequence utilizing chromatin immunoprecipitation (ChIP) assay. Results demonstrated that c-Rel was recruited onto the PELP1 promoter sequence between −576 to −585 bp (Fig. 3*J*), and there was a drastic increase in the c-Rel binding on treatment with LPS. In addition, to validate the direct confinement of c-Rel onto the PELP1 promoter, we performed EMSA using oligonucleotide probes spanning the c-Rel consensus region. Results showed the formation of DNA–protein complexes in the reactions where WT probes were used, whereas there was no binding when the mutant probes were utilized (Fig. 3*K*). The observed complex was significantly enhanced when the LPS-treated protein extracts were incubated with WT probes. We confirmed the specificity of c-Rel binding to the oligo probes by performing super shift assay where we observed the disappearance of transcription factor–DNA complex when the reaction was incubated with c-Rel–specific antibody (Fig. 3*L*). To confirm c-Rel is the specific transcription factor that regulates LPS-mediated PELP1 expression, we used c-Rel–specific inhibitor pentoxifylline (Ptx) (32). Results showed a significant decrease in the LPS-mediated induction of PELP1 upon pretreatment with Ptx at protein, mRNA, and PELP1 promoter activity levels (Fig. 4, *A*–*C*). These results were further confirmed by the finding that there was a notable decrease in the LPS-mediated induction of PELP1 protein, mRNA, and PELP1 promoter activity with c-Rel–specific siRNA-transfected RAW 264.7 cells when compared with control siRNA-transfected cells (Fig. 4, *D*–*F*). These experiments revealed the mechanistic and functional significance of c-Rel in the regulation of PELP1 at transcriptional level during an inflammatory response.

### GM-CSF—a new molecular target of PELP1

**Figure 5:**
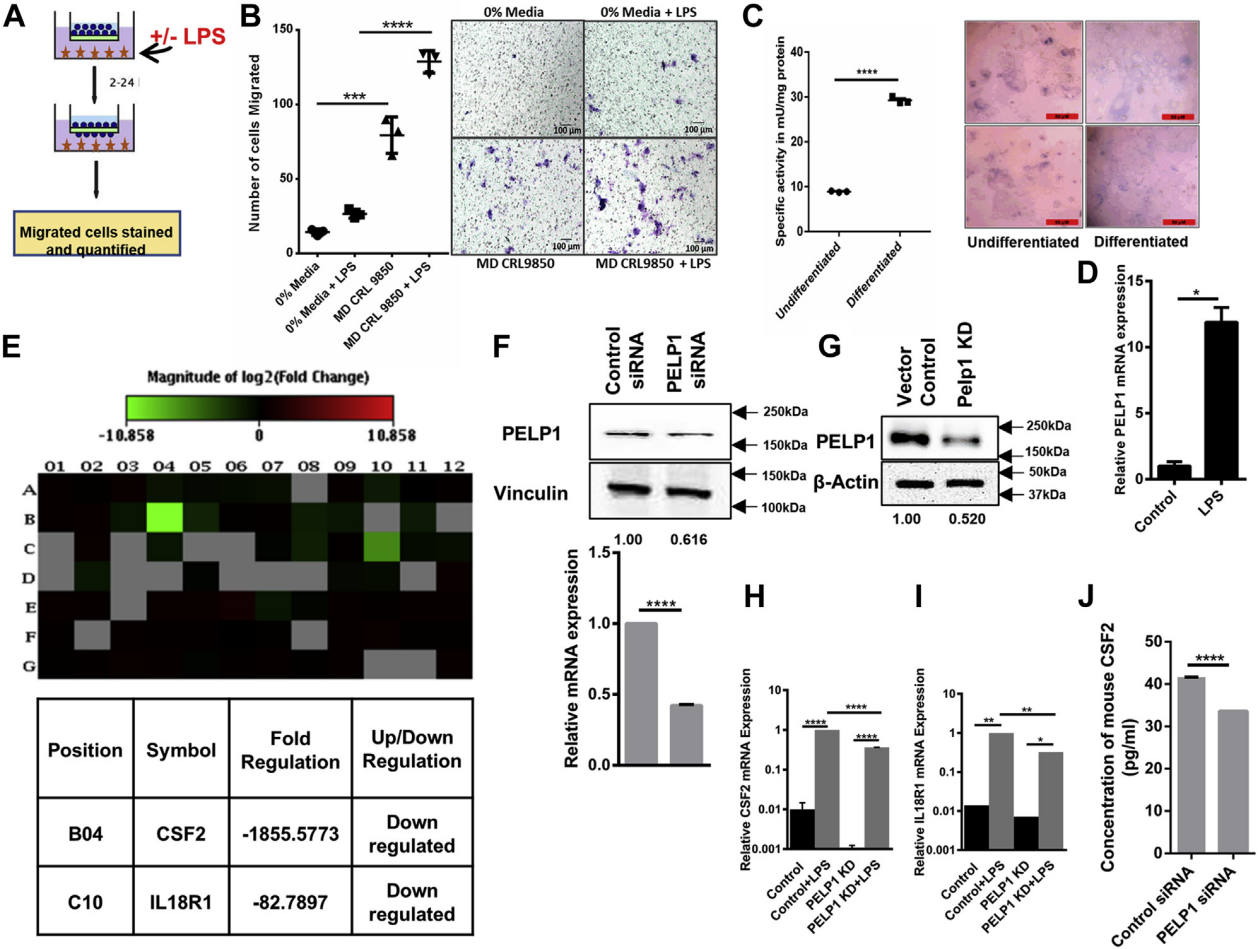
**GM-CSF—a new molecular target of PELP1.***A*, the schematic represents coculturing of Caco2 cells with MD-CRL9850 (with/without LPS) in a transwell migration apparatus. *B*, *left*, scattergram represents the number of migrated Caco2 cells when cocultured with 0% media, 0% media with LPS, LPS (1 μg/ml) treated/untreated MD-CRL 9850 cells. *Right*, representative phase contrast images of migrated Caco2 cells. *C*, *left*, specific activity of alkaline phosphatase (mU/mg of protein) in undifferentiated and differentiated Caco2 cells. *Right*, representative phase contrast images of Caco2 cells. *D*, quantitative RT (qRT)–PCR analysis of PELP1 in LPS-treated and untreated MD-CRL9850. *E*, heat map showing the expression of 84 key regulatory genes in mouse IL-6/STAT3 inflammatory signaling pathway determined by using PELP1 knocked down RAW 264.7 lysate. *Green* and *red* color backgrounds indicate the downregulation and upregulation of specific gene expressions, respectively. The position, gene symbol, and fold change of genes downregulated in PELP1 knockdown condition were represented below the heat map. *F*, Western blot and qRT–PCR images that confirmed PELP1 KD when RAW 264.7 cells were transfected with PELP1 siRNA. *G*, Western blot that confirmed PELP1 KD by shRNA lentiviral particles in RAW 264.7. *H*–*I*, validation of GM-CSF and IL18R1 expression by qRT–PCR using RNA from RAW 264.7 PELP1 KD clone with and without LPS induction. *J*, cell culture supernatants from PELP1 siRNA-transfected RAW 264.7 cells were collected after 48 h of transfection and assessed for GM-CSF concentration (pg/ml) using ELISA. GM-CSF, granulocyte–macrophage colony-stimulating factor; IL, interleukin; LPS, lipopolysaccharide; PELP1, proline, glutamic acid, and leucine-rich protein 1; STAT3, signal transducer and activator of transcription 3.

Before identifying the downstream molecular targets that might be regulated by PELP1 signaling, we wanted to confirm whether LPS stimulation can induce cellular transformation or not. We constructed a macrophage-epithelial coculturing system using MD-CRL9850, a human macrophage cell line and Caco2, a human colon adenocarcinoma cell line. Caco2 has a peculiar property of spontaneous differentiation into intestinal epithelial cells when cultured for 2 to 3 weeks after attaining confluence (33). Differentiated Caco2 cells exhibit characteristics like polarized columnar morphology (34), increased levels of digestive brush border enzymes, higher alkaline phosphatase activity, and increased membrane potential (35). This property of Caco2 cells has been utilized by researchers around the world for decades to understand cellular transformation by various chemical drugs. In this study, this ability of Caco2 was exploited for coculturing with MD-CRL9850 cell line. When differentiated Caco2 cells were seeded on top of an insert, followed by addition of LPS-stimulated/unstimulated MD-CRL9850 cells in the bottom of the Trans-well Boyden chamber as depicted in Figure 5*A*, we observed an increase in the number of migrated Caco2 cells in the LPS-stimulated MD-CRL9850 added chamber (Fig. 5*B*). In Figure 5*C*, the morphology of differentiated Caco2 cells and their alkaline phosphatase activity were compared with undifferentiated cells. Figure 5*D* represents an increase in the relative PELP1 mRNA levels in LPS-stimulated MD-CRL9850 when compared with unstimulated cells. In brief, these results showed that LPS-induced PELP1 expression in macrophages aided epithelial cell migration. This observation was correlated with our clinical experimental results showing increased PELP1 expression in inflammatory tissues of appendicitis and colitis compared with normal tissues.

To determine the downstream molecular targets of PELP1 that can furnish an inflammatory signaling response, we performed qRT–PCR expression profiling of 84 key regulatory genes connected with inflammatory pathway (mouse IL6/STAT3 signaling pathway PCR array) using RNA samples collected from control siRNA and PELP1 siRNA-transfected RAW 264.7 cells. We found CSF-2 and IL18R1 as the putative molecular targets that were substantially downregulated in PELP1 siRNA samples as compared with control siRNA (Fig. 5, *E* and *F*). We further validated CSF-2 and IL18R1 expression along with LPS induction using individual qRT–PCR assay and observed that mRNA was significantly downregulated in RAW 264.7 PELP1 knockdown (KD) clone (Fig. 5, *G*–*I*). Between CSF-2 and IL18R1, we chose to study CSF-2 as a potential target of PELP1 because of its proven role in cancer progression and as a secretory paracrine mediator. CSF-2 is a monomeric glycoprotein also termed as GM-CSF, secreted by various immune cells, and acts as an inflammatory cytokine (36). We estimated the secretory CSF-2 levels in cell culture supernatants by ELISA, and the results confirmed a substantial reduction in its expression with PELP1 downregulation (Fig. 5*J*). With all these aforementioned experiments, we assumed that PELP1, through the regulation of GM-CSF, might influence an inflammatory response.

### Regulation of GM-CSF by PELP1

**Figure 6:**
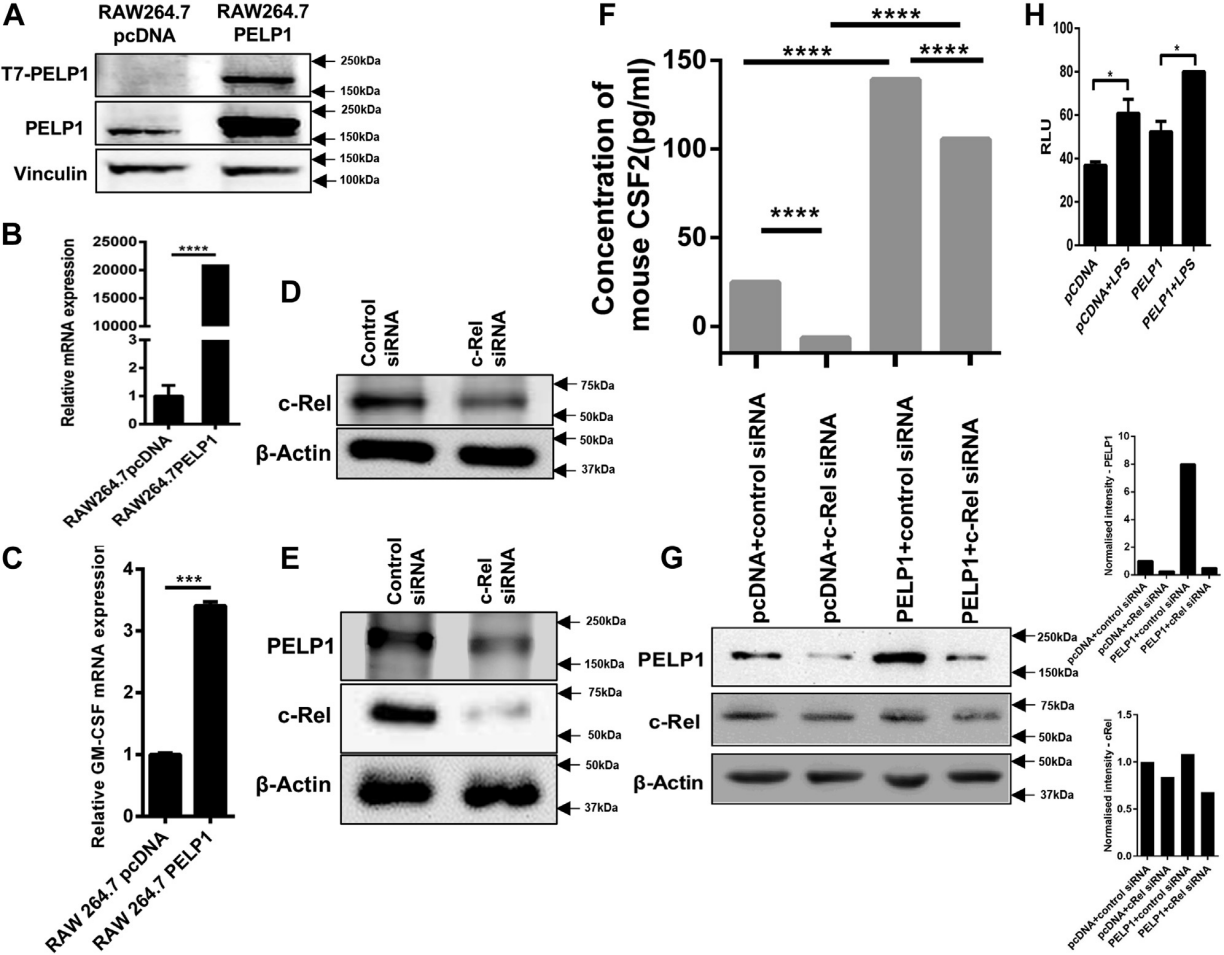
**Regulation of GM-CSF by PELP1.***A* and *B*, represent PELP1 overexpression by Western blot and quantitative RT–PCR in RAW 264.7 clones. *C*, GM-CSF expression in RAW 264.7-PELP1 overexpression clones by quantitative RT–PCR analysis. *D* and *E*, Western blots showing reduced expression of c-Rel when transfected with two different c-Rel siRNAs having different target sequences in RAW 264.7. *E*, also showed downregulation of PELP1 in c-Rel siRNA-transfected RAW 264.7 cells. *F*, ELISA analysis of GM-CSF expression regulated by PELP1 when transfected with c-Rel siRNA in RAW 264.7-PELP1 clones. *G*, Western blot and its quantification images representing PELP1 downregulation when transfected with c-Rel siRNA in RAW 264.7-PELP1 clones. *H*, inflammasome assay on RAW 264.7 PELP1 overexpression cells. Clones were treated with LPS (1 μg/ml for 4 h), and luminescence was measured after 60 min of incubation with Caspase-Glo-1 reagent. GM-CSF, granulocyte–macrophage colony-stimulating factor; LPS, lipopolysaccharide; PELP1, proline, glutamic acid, and leucine-rich protein 1.

In order to confirm the functional significance of PELP1 in regulation of GM-CSF, we constructed PELP1 overexpressing clones in RAW 264.7 cells (Fig. 6, *A* and *B*). The expression of GM-CSF was positively correlated with the overexpression of PELP1 at mRNA level by qRT–PCR analysis (Fig. 6*C*). When RAW 264.7 cells were transfected with two different c-Rel siRNAs having different target sequences, reduced expression of c-Rel was observed (Fig. 6, *D* and *E*). Downregulation of PELP1 was also observed in c-Rel siRNA-transfected RAW 264.7 cells (Fig. 6*E*). When PELP1 overexpression clones of RAW 264.7 cells were transfected with c-Rel siRNA, the results showed that the expression of GM-CSF was downregulated in PELP1 overexpression clones (Fig. 6, *F* and *G*). This proved the role of PELP1 in the regulation of GM-CSF. Inflammasome formation is a key event in the innate immune response that involves activation of caspase-1. When RAW 264.7 PELP1 overexpression clones were treated with LPS, we found that overexpression of PELP1 stimulated inflammasome formation, which in turn explained the importance of PELP1 as a key component of inflammatory responses (Fig. 6*H*).

### GM-CSF—a specific mediator of cellular transformation *via* paracrine signal

**Figure 7:**
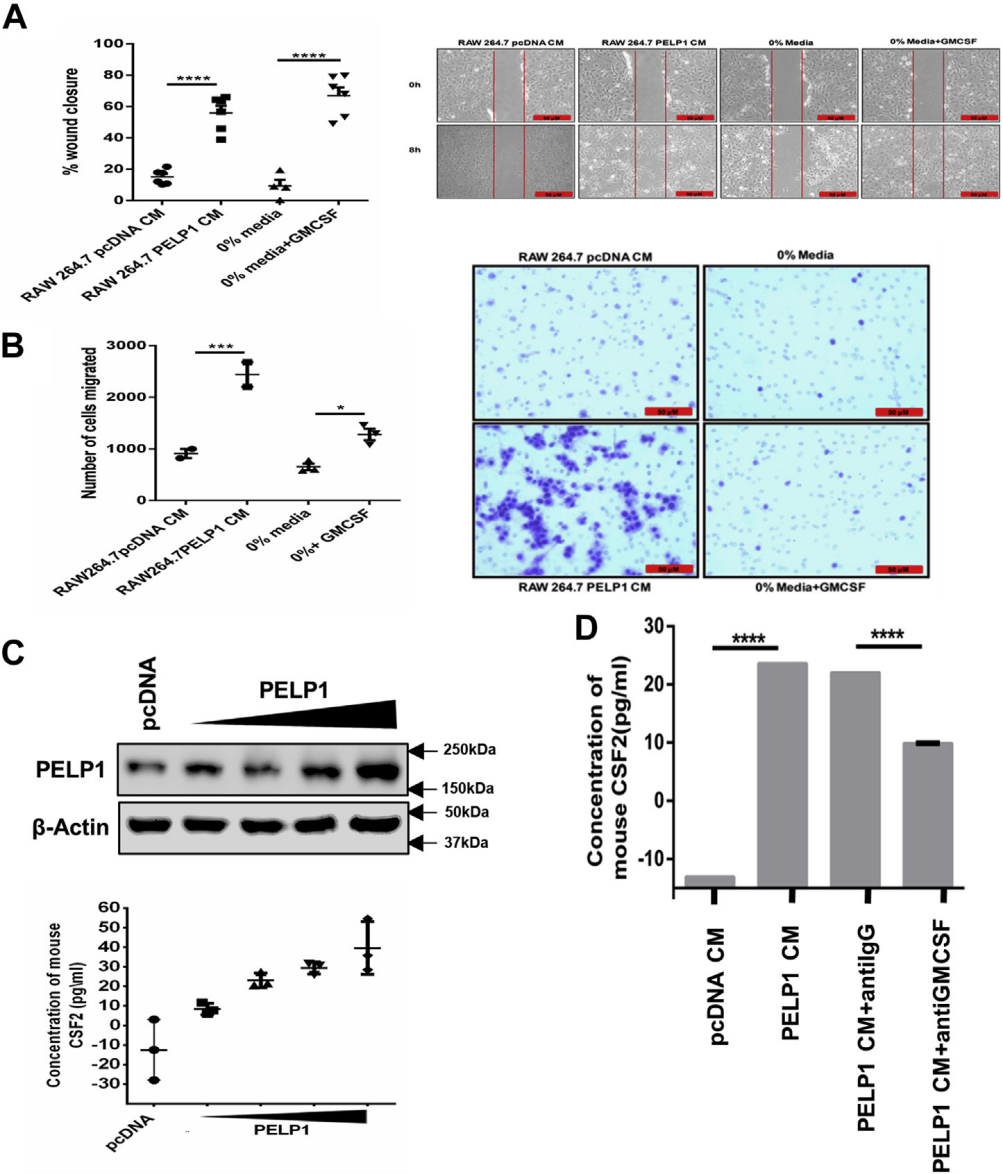
**GM-CSF—a specific mediator of cellular transformation in a paracrine manner.***A*, wound healing assay of NIH3T3 cells incubated with conditioned media (CM) collected from RAW 264.7-PELP1 overexpression clones along with 0% serum media and GM-CSF pure protein as controls. The *left panel* depicts percentage of wound closure, and the *right panel* consists of representative images of wound closure. *B*, transwell migration assay of NIH3T3 (in the *upper chamber*) incubated with CM collected from RAW 264.7-PELP1 overexpression clones (in the *bottom chamber*). The *left panel* depicts the number of migrated cells, and the *right panel* shows representative images of migrated NIH3T3 cells captured by phase contrast microscope connected to a camera. *C*, ELISA to confirm increase in expression of GM-CSF along with increase in PELP1 expression in RAW 264.7 cells. Increasing concentration of PELP1 expression plasmid was transfected in RAW 264.7 cells, and the CM collected were concentrated to measure the CSF-2 concentration. Western blot showed the increased expression of PELP1 in RAW 264.7 cells transfected with pcDNA vector and increasing concentration of PELP1 expression plasmid. *D*, ELISA to confirm GM-CSF depletion in RAW 264.7-PELP1 clones. The depletion was achieved by incubating CM with anti GM-CSF antibody for 1 h at 37 °C followed by adding protein A/G beads for 2 to 4 h at 4 °C. GM-CSF, granulocyte–macrophage colony-stimulating factor; PELP1, proline, glutamic acid, and leucine-rich protein.

GM-CSF is reported to be involved in tumor progression in different cancers in an autocrine manner or a paracrine manner (37, 38). To identify whether GM-CSF secreted from PELP1 overexpression clones of RAW 264.7 cells has any impact on cancer transformation, we used concentrated and filter-sterilized conditioned media (CM) collected from PELP1 overexpression clones of RAW 264.7 cells for functional studies. CM obtained from PELP1 overexpression clones had increased the migration potential in NIH3T3 when compared with pcDNA CM (Fig. 7*A*). In addition, transwell cell migration assay further confirmed the migration-promoting ability of CM collected from PELP1 overexpression clones on NIH3T3 cells (Fig. 7*B*). In order to show that the levels of GM-CSF expression directly correlate with the expression of PELP1, increasing concentrations of PELP1 expression plasmid were transfected in RAW 264.7 cells. The CM collected from transfected RAW 264.7 cells were concentrated, and the CSF-2 concentration was measured using ELISA. This experiment showed that GM-CSF expression concomitantly increased with PELP1 expression (Fig. 7*C*).

**Figure 8:**
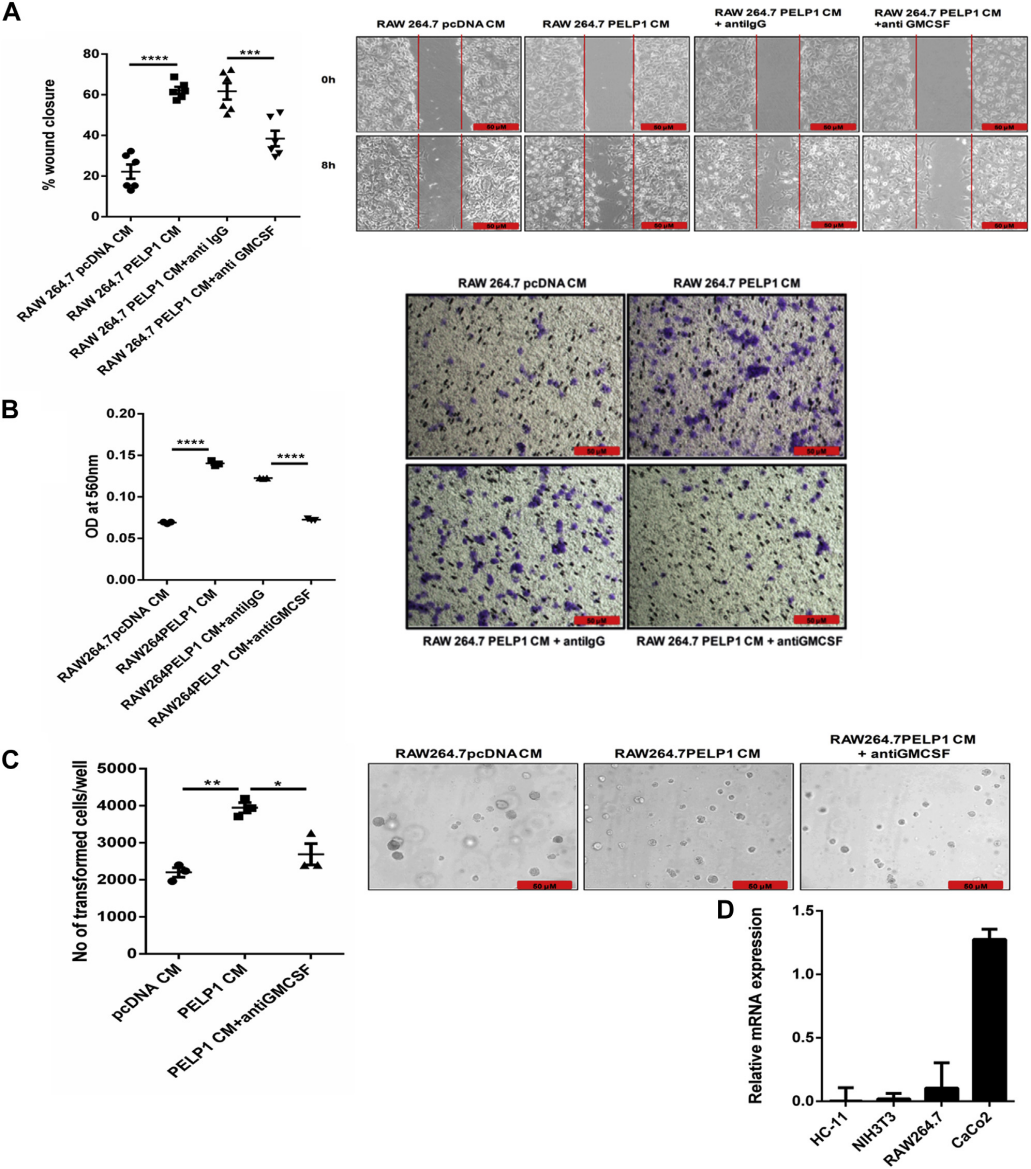
**GM-CSF—a specific mediator of cellular transformation in a paracrine manner.***A* and *B*, wound healing and transwell migration assay of NIH3T3 cells incubated with conditioned media (CM)/GM-CSF depleted CM collected from RAW 264.7-PELP1 overexpression clones. The *left panels* represent percentage of wound closure and the number of migrated cells; the *right panels* show representative images of wound healing and transwell migration assay of NIH3T3 cells, respectively. *C*, soft agar colony formation assay; *Left*, the total number of transformed NIH3T3 cells/well when incubated with CM/GM-CSF–depleted CM collected from RAW 264.7-PELP1 overexpression clones for 1 to 2 weeks. *Right*, representative images captured by phase contrast microscope connected to a camera. *D*, quantitative RT–PCR analysis of CSF-2–specific receptor subunit alpha (CSFRa) expression on various cell lines. GM-CSF, granulocyte–macrophage colony-stimulating factor.

To validate GM-CSF present in CM as the factor responsible for the migration potential of NIH3T3, we performed functional assays utilizing GM-CSF–depleted CM. The depletion of GM-CSF was achieved by incubating the CM with GM-CSF neutralizing antibody followed by a pull down of the antibody–cytokine complex utilizing protein A/G beads, which was confirmed by ELISA (Fig. 7*D*). Results from wound healing assay showed a reduction in the migration rate, and transwell cell migration assay showed a reduction in the number of migratory cells in the GM-CSF–depleted CM added to NIH3T3 cells (Fig. 8, *A* and *B*). This was further confirmed with soft agar colony formation assay (Fig. 8*C*). Since the impact of PELP1 on inflammation-induced tumorigenesis is mediated by GM-CSF signaling, we also confirmed the expression of GM-CSF–specific receptor subunit CSF-2 receptor subunit alpha on various cell lines by RT–PCR analysis (Fig. 8*D*).

### Feedback regulation of PELP1 expression in cancer

**Figure 9:**
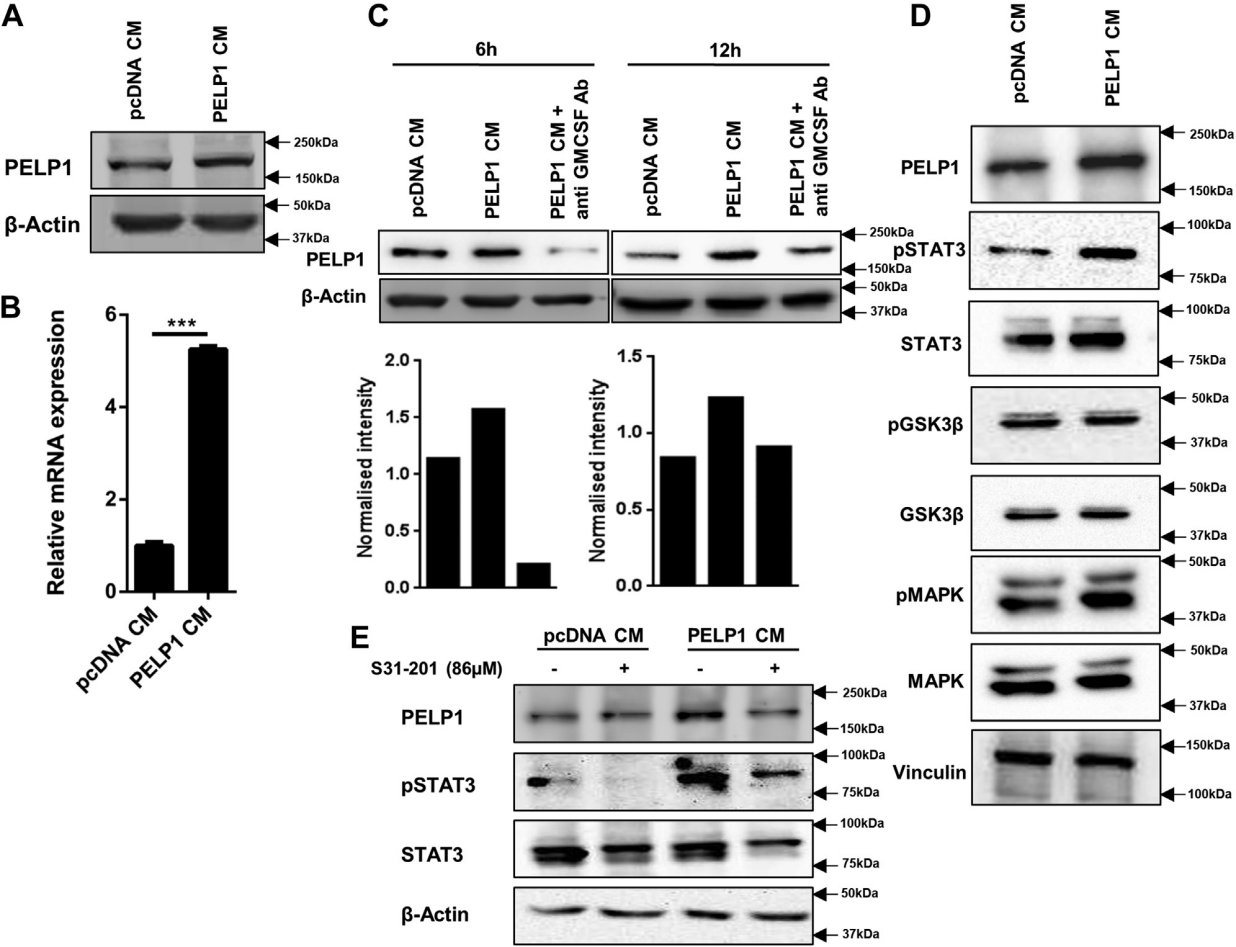
**Feedback regulation of PELP1 expression in cancer.***A* and *B*, Western blot and quantitative RT–PCR analysis of PELP1 expression in HC11 cells incubated with CM collected from RAW 264.7-PELP1 clones for 6 h. β-Actin was used as control. *C*, Western blot analysis of PELP1 expression in HC11 cells when incubated with CM/GM-CSF–depleted CM collected from RAW 264.7-PELP1 clones for 6 and 12 h time intervals. *D*, Western blot analysis of various cytokine-mediated signaling pathway proteins along with PELP1 in HC11 cells incubated with CM collected from RAW 264.7-PELP1 clones. *E*, Western blot analysis of HC11 cells pretreated with S31-201 inhibitor (86 μM) followed by CM treatment collected from RAW 264.7-PELP1 clones. Actin and total STAT3 as loading controls. CM, conditioned media; GM-CSF, granulocyte–macrophage colony-stimulating factor; PELP1, proline, glutamic acid, and leucine-rich protein 1.

As the transformation potential of CM from PELP1 overexpression clones of RAW 264.7 cells was evident from NIH3T3-cell transformation assays, we questioned whether PELP1 expression could be regulated in a feedback manner. To explain feedback regulation of PELP1 in inflammation-driven cancers, we incubated mouse mammary epithelial cell line, HC11, with CM collected from PELP1 overexpression clones of RAW 264.7 cells. The results indicated that the HC11 incubated with CM from PELP1 overexpression clones showed an increase in PELP1 expression compared with CM from pcDNA clone (Fig. 9, *A* and *B*). To elucidate that the GM-CSF present in the CM might be specifically responsible for the phenomenon, we performed GM-CSF depletion assay. The results explained that the PELP1 induction was inhibited when the cells were incubated with GM-CSF–depleted CM (Fig. 9*C*).

To elucidate the mechanism of signal transmission of GM-CSF to overexpress PELP1, we analyzed the expression of various cytokine-mediated signaling molecules in CM-stimulated HC11 cells. In this assay, we observed a significant difference in the expression of STAT3 when HC11 cells were incubated with CM collected from PELP1 overexpression clones than the cells that were incubated with CM from pcDNA control clones (Fig. 9*D*). To support this, we preincubated the HC11 cells with STAT3-specific inhibitor S31-201, at a minimum inhibitory concentration of 86 μM and then with CM from PELP1 overexpression clones. In this experiment, we observed inhibition of pSTAT3 along with downregulation of PELP1 expression (Fig. 9*E*). This result explained that Janus kinase (JAK)/STAT signaling pathway plays crucial role in GM-CSF–mediated PELP1 overexpression in HC11.

### PELP1 expression levels are elevated in clinical inflammatory disease conditions and its connection in human normal–inflammation–tumor spectra

Various evidences indicate that chronic inflammation renders a microenvironment which contributes to oncogenesis (39). Despite the existence of a large amount of literature linking PELP1 with human cancer, evidence in support of a contributing role of PELP1 in inflammatory responses still remains obscure. We were intrigued to identify the role of PELP1 in inflammation as a potential responsive gene. To test our hypothesis, two different models of acute and chronic inflammation, a human acute appendicitis model and a mouse dextran sulfate sodium (DSS)–induced colitis model, were studied.

**Figure 10:**
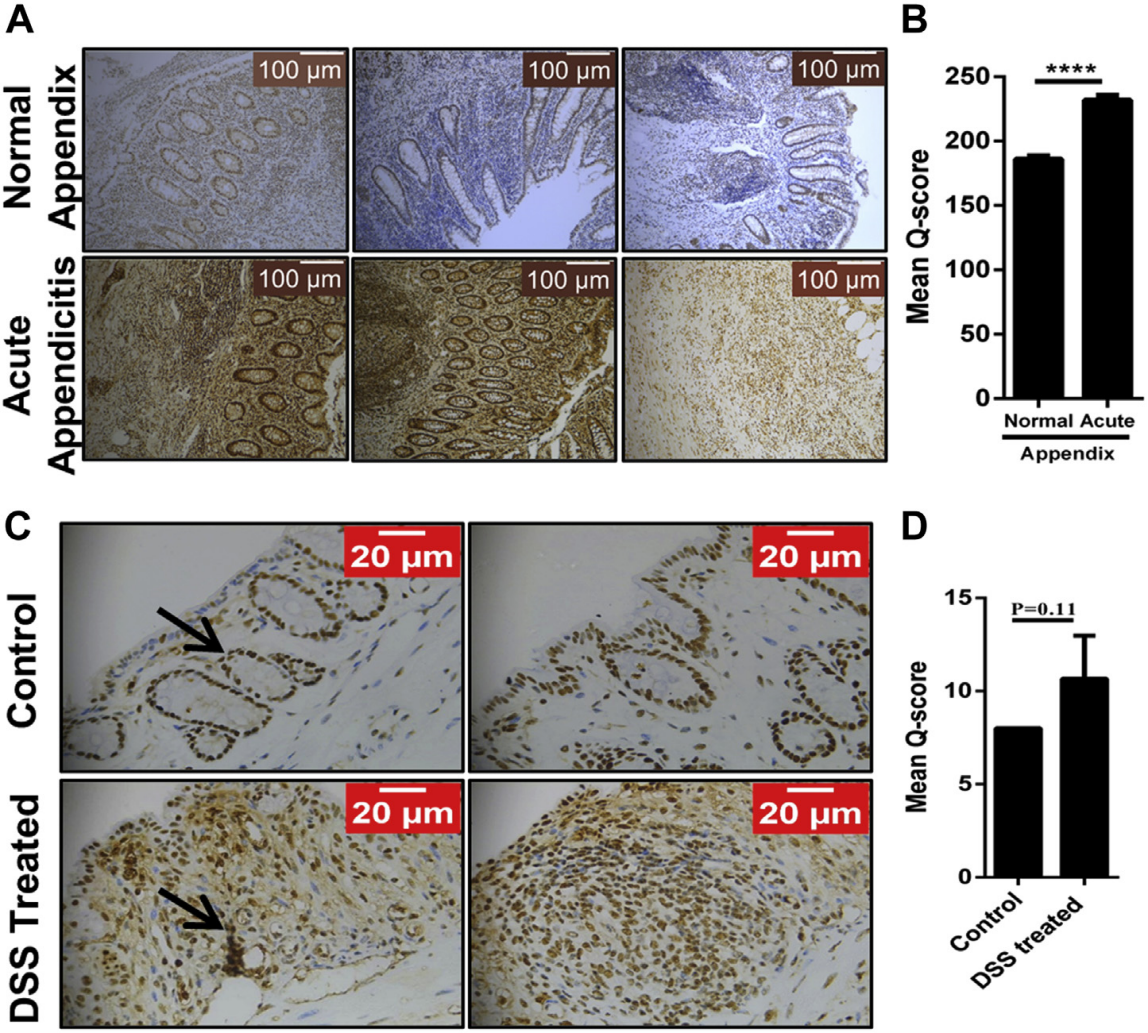
**PELP1 expression in inflammatory disease conditions.***A*, IHC analysis of PELP1 in normal and acute appendicitis specimen (N = 69) showing higher PELP1 positivity in acute appendicitis in contrast to normal appendix. *B*, the mean Q score of normal appendix *versus* acute appendicitis was plotted; a strong significance of 0.0005 was observed. *C*, IHC analysis of PELP1 in control and DSS-induced colitis model specimen (N = 3) showing higher PELP1 positivity in DSS-induced model than the control. *D*, the mean Q score of control *versus* DSS-treated model were plotted, a *p* value of 0.11 was observed. DSS, dextran sulfate sodium; IHC, immunohistochemistry; PELP1, proline, glutamic acid, and leucine-rich protein 1.

Appendicitis is an acute prevalent inflammatory condition of the vermiform appendix caused by purulent microflora characterized by infiltration of various immune cells (40), and hence, it is considered as an ideal model to study inflammation. To decipher the role of PELP1 in inflammatory diseases, we screened 69 paired human samples (N = 69) of normal and acute appendicitis. IHC staining for PELP1 on these tissue specimens showed an intense nuclear positivity in the epithelial and myoepithelial cells of the mesothelial layer, and the infiltrating neutrophils showed higher expression of PELP1 compared with the normal tissue sections (Fig. 10*A*). The Mean Q score of PELP1 was found to be 232.03 ± 31.55, as compared with the adjacent uninvolved appendix, which had a mean Q score of 186.09 ± 21.84, with a *p* value of 0.0005 (Fig. 10*B*). This is a clear evidence of high PELP1 expression in acute appendicitis.

To substantiate our findings, a simple, robust, and widely used DSS-induced colitis model was utilized because of its resemblances with human ulcerative colitis (41, 42). The DSS-treated and control sections were obtained from Dr Anita Sjolander from Lund University, and the tissue sections were prepared as described earlier (43, 44). When the DSS-treated and control sections were stained for PELP1, the results showed an increase in PELP1 expression in DSS-treated slides compared with the controls (Fig. 10, *C* and *D*).

**Figure 11:**
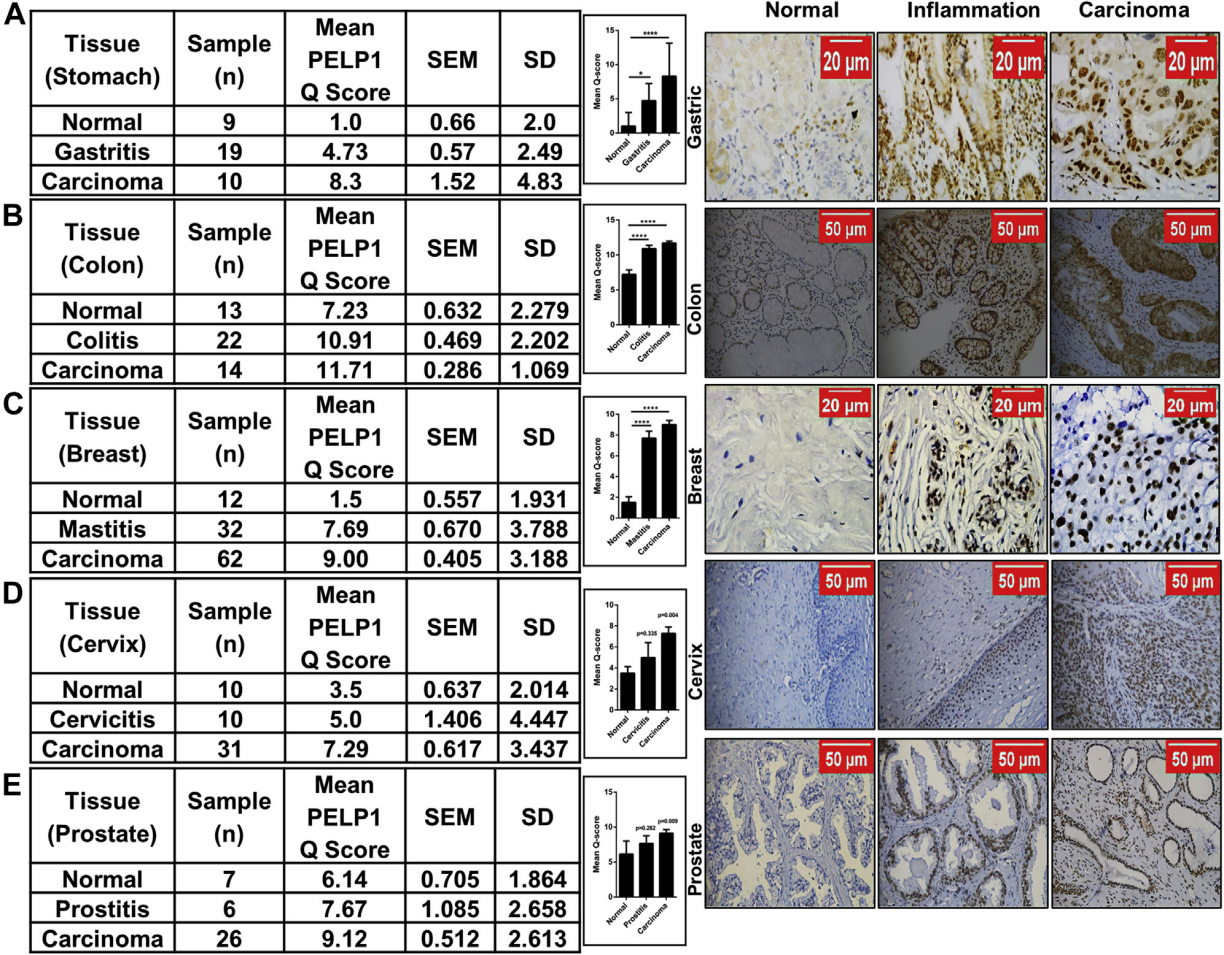
**PELP1 expression in human normal–inflammation–tumor spectra.***A*–*E*, *left*, statistical analysis of IHC staining of PELP1 in normal–inflammation–tumor progression models of gastric, colon, breast, cervix, and prostate cancer disease spectra showing higher PELP1 positivity in a progressive manner from normal to cancer. The histograms represent mean Q score of PELP1 expression in normal, inflammation, and cancer specimens of respective tissues. *Right*, representative images of normal–inflammation–tumor progression models of gastric, colon, breast, cervix, and prostate cancer disease spectra. IHC, immunohistochemistry; PELP1, proline, glutamic acid, and leucine-rich protein 1.

In continuation to the aforementioned experiments, to identify the clinical significance of PELP1 and its connection in inflammation-associated cancer progression, we examined the expression of PELP1 in various progression models of normal–inflammation–cancer disease spectra in which the cancer mainly arises from a pre-existing chronic inflammatory microenvironment. The IHC results clearly indicated a gradual increase in PELP1 expression in gastric, colon, breast, cervical, and prostate cancer disease models (Fig. 11, *A*–*E*). These data gave us a clue that PELP1 is one of the prime molecules that may function as a connecting link between inflammation and cancer.

### Demonstrating the role of PELP1 as a connecting link in inflammation-driven cancer progression *in vivo* animal models

**Figure 12:**
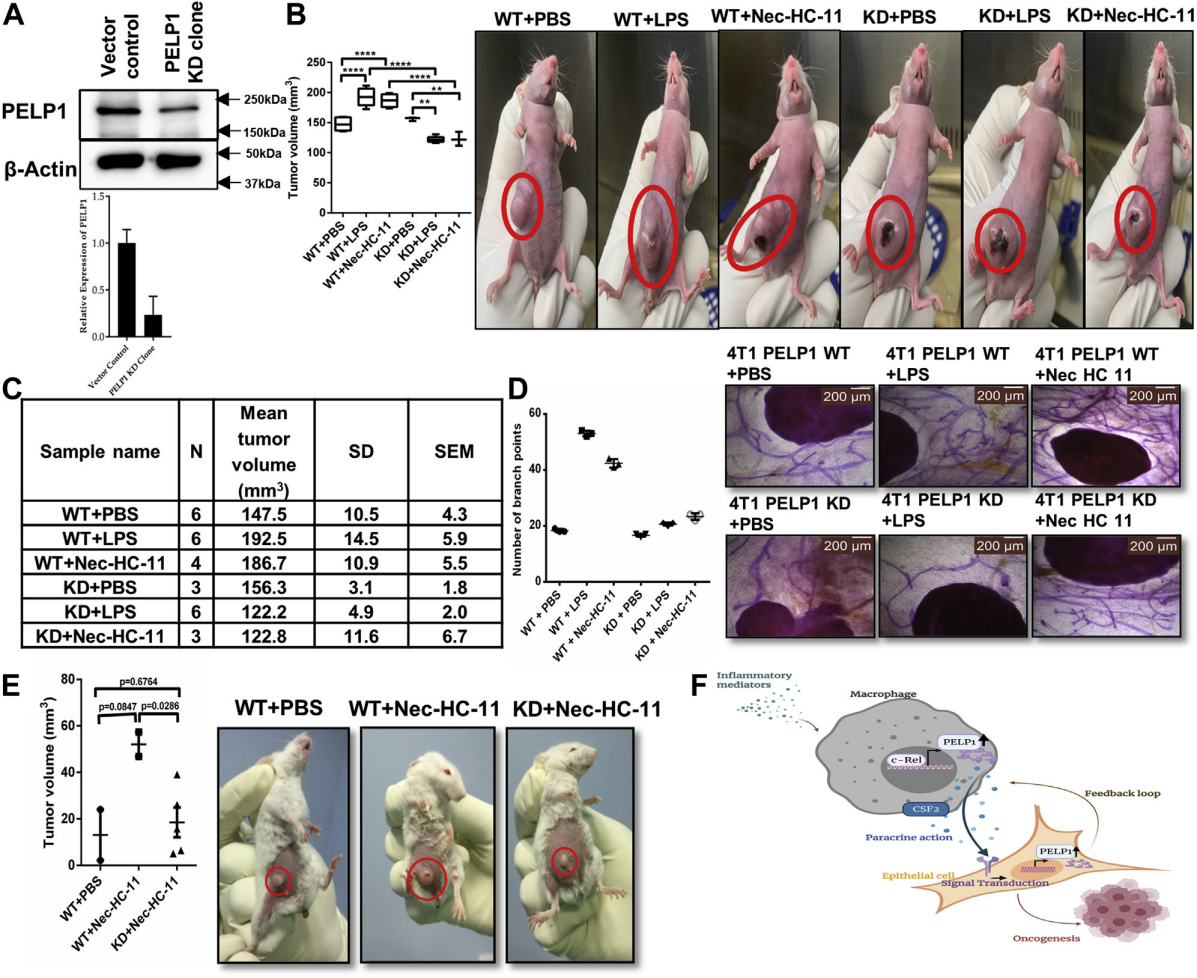
**Demonstrating the role of PELP1 as a connecting link in inflammation-driven cancer progression *in vivo* animal models.***A*, Western blot (*top*) and quantitative RT–PCR (*bottom*) analysis of PELP1 expression in PELP1 knocked down 4T1 clones. *B*, *left*, tumor volume measured after 2 to 3 weeks in female nude mice injected with or without LPS or necrotic HC11 lysate (Nec-HC11) along with 4T1 WT and PELP1 knockdown (KD) cells injected into the mice mammary fat pads. *Right*, representative images of mice with tumors. *C*, statistical analysis table with N, mean tumor volume, SD, and SEM. *D*, *left*, scattergram showing the number of mammary gland ductal branching in PBS control, LPS, or Nec-HC11 stimulated 4T1-WT or PELP1 KD injected mice. *Right*, representative images of mice mammary gland whole mounts. *E*, *left*, tumor volume measured after 2 to 3 weeks in female BALB/c mice injected with or without necrotic HC11 lysate (Nec-HC11) along with 4T1 clones injected into the mice mammary fat pads (N = 2 for WT + PBS and WT + Nec-HC11; N = 5 for KD + Nec-HC11). *Right*, representative images of mice with tumors. *F*, the overall depiction of mechanism of PELP1 mediating signaling mechanism in inflammation-driven cancer progression. LPS, lipopolysaccharide; PELP1, proline, glutamic acid, and leucine-rich protein 1.

To emphasize the importance of PELP1 *in vivo* as a link between inflammation and cancer, we generated PELP1 KD clones in 4T1, a murine mammary carcinoma cell line (Fig. 12*A*), and then injected them into female nude mice after stimulating with LPS/necrotic HC11 lysate. We noticed an increase in tumor volume in LPS or Nec-HC11–stimulated mice that were injected with WT cells, whereas it was significantly reduced in LPS or Nec-HC11–stimulated mice that were injected with KD cells (Fig. 12, *B* and *C*). Notably, a prominent decrease in mammary gland ductal branching was observed in LPS-stimulated or Nec-HC11–stimulated mice that were injected with KD cells (Fig. 12*D*).

To confirm the effect of PELP1 KD on tumor formation during inflammation, we injected 4T1 clones into the mammary fat pads, stimulated with Nec-HC11 in female BALB/c (immunocompetent) mice. Once again, we noticed a reduced tumor volume in Nec-HC11–stimulated mice that were injected with KD cells when compared with WT cells as shown in Figure 12*E*. Our results from *in vivo* studies established a strong proof showing PELP1 as one of the critical molecules that may function as a link between inflammation and cancer.

## Discussion

Various reports support the fact that chronic inflammation can increase the risk of developing many types of cancer and is considered as an important characteristic in the hallmarks of cancer (45). Till date, numerous signaling molecules have been identified whose primary function is to modulate inflammatory response, but any alteration in their expression or activity results in cancer formation (46, 47). We focused our attention on the causative role of PELP1 in inflammation and to identify its novel mediators responsible for inflammation-associated cancer progression. A recent study explained that cytoplasmic PELP1 induces secretion of inflammatory cytokines into a protumorigenic microenvironment through NF-κB, which promotes tumor initiation (25). In our study, for the first time, we illustrated the mechanism of transcriptional regulation of PELP1 during inflammation and identified the downstream molecular targets of PELP1 responsible for inflammation to tumor transformation.

To uncover PELP1 as an inflammatory responsive gene, we framed a series of experiments systematically with a combination of Western blot and qRT–PCR analysis exploiting macrophages as the model for our study. This is because the macrophages are the first cells that are recruited upon infection or tissue injury and function as a prime source of various growth factors and secretory cytokines for distinct cell types in the local microenvironment (48). Macrophages are recruited not only to sites of inflammation but also by cancer cells (49), thus playing a major role in inflammation-driven cancers by releasing cytokines that promote cancer cell survival and proliferation in the tumor microenvironment (50). We identified that PELP1 was significantly upregulated in a wide range of macrophage cell lines upon induction with inflammatory stimulus. The various *in vivo* experiments further strengthen the key point of universal relevance in increased PELP1 expression during inflammation. The observation of PELP1 induction was convincing even in the case of inflammation induced by cell injury, termed as sterile inflammation (28). In addition, experiments using treatment with NSAIDs had further proved the importance of PELP1 as a crucial player in inflammation and proposes plausible interventions.

The transcriptional regulation of PELP1 results from an interesting observation that PELP1 promoter was induced upon treatment with inflammatory cytokines. This prompted us to identify the transcription factor responsible for regulation of PELP1 at the promoter level. We demonstrated that c-Rel, an NF-κB family transcription factor, has the potential to modulate PELP1 expression at the gene level through various *in silico*, *in vitro*, and *in vivo* experiments. With this in mind, we performed siRNA-mediated KD studies and experiments using protein-specific inhibitors, which served as an additional proof to confirm the role of c-Rel in PELP1 induction during inflammation. To our knowledge, this is the first report to describe c-Rel–specific transcriptional regulation of PELP1 in inflammation. According to our findings, in the presence of inflammatory stimulus, macrophages get activated and result in the production of PELP1 protein through the transcriptional function of c-Rel.

The macrophage–epithelial cell coculturing system paved the way to evaluate the transformation potential of activated macrophages on epithelial cells in a paracrine manner. The qRT–PCR microarray for key regulatory genes implicated during inflammation using PELP1 KD samples exposed GM-CSF and IL18R1 as the molecular targets of PELP1. Between GM-CSF and IL18R1, we explored GM-CSF as a potential target, as it is a secretory cytokine in nature with the capacity to transform adjacent epithelial cells in a paracrine manner and whose role is well established in progression of a few cancer types (36, 37). CSF-2, also termed as GM*-*CSF, is a monomeric glycoprotein secreted by macrophages, T cells, mast cells, NK cells, endothelial cells, and fibroblasts and functions as a cytokine that is reported to be involved in tumor progression in different types of cancers in an autocrine manner or a paracrine manner. In our study, we have shown abundance of CD-68–positive macrophages in the inflamed breast tissues adjacent to the breast tumor confirming their role in tumor progression in companion with initiated epithelial cells. In our study, the expression of GM-CSF was correlated with PELP1 overexpression and observed to be functionally regulated through PELP1. But the molecular mechanism of GM-CSF upregulation by PELP1 was still imprecise and needed to be explored. Furthermore, we showed that GM-CSF was responsible for migration and cellular transformation ability *in vitro* in a paracrine manner. Also, GM-CSF released by the macrophages upon inflammatory stimulus through c-Rel-PELP1 signaling in turn induces the expression of PELP1 in the neighboring epithelial cells in a feedback manner. Next, we were fascinated to study the signaling mechanism of GM-CSF transduction to overexpress PELP1 in epithelial cells. In general, the transduction of cytokine signaling can be mediated mainly through three different pathways that are JAK/STAT, Akt-phosphoinositide 3-kinase, and extracellular signal–regulated kinase–mitogen-activated protein kinase (MAPK) signaling pathways (51). In this study, the mechanism through which GM-CSF exerted its action was observed to be by JAK/STAT pathway. However, the complete mechanism of signal transduction of GM-CSF to overexpress PELP1 in epithelial cells needs to be explored. The overall mechanism of PELP1-mediating inflammation-driven cancer progression is depicted in Figure 12*F*. The clinical relevance for PELP1 induction during inflammation was brought out from our primary studies like human acute appendicitis (40) and DSS-induced mouse chronic ulcerative colitis (41) models. Furthermore, our analysis of PELP1 expression in various cancers that arise from a pre-existing chronic inflammatory background in a progression model of normal–inflammation–cancer disease spectrum showed a progressive increase in PELP1 expression in gastric, colon, breast, cervical, and prostate cancers. This analysis can be better extended to other inflammation-driven cancers like pancreas, oral, and so on, which increases the potential of PELP1 as a clinical marker. The *in vivo* study on athymic and BALB/c mice presented strong evidence linking PELP1 in inflammation-directed cancer progression.

In conclusion, our study has presented PELP1 as an essential molecule for a number of important cellular processes, and its dysfunction is associated with various diseases, mainly cancer. It is worth studying the involvement of different signaling molecules in PELP1 regulation and molecules regulated by PELP1 signaling. Understanding the regulatory mechanisms of PELP1 could help making better therapeutics to reduce the occurrence of several cancers associated with inflammation. In the current scenario, our work would present a platform emphasizing the novel role of PELP1 as a key oncogene in inflammation-driven cancers. We strongly believe that exploring the drug targets, active pockets of the protein, and its interacting partners along with the dynamic interplay among immune cells, cancer cells, and its associated heterogenic tumor microenvironment will open up new horizons to create its own space in cancer therapy.

## Experimental procedures

### Cell lines and reagents

Murine macrophage cell line RAW 264.7 was procured from NCCS and was maintained in Dulbecco's modified Eagle's medium (DMEM) (MP Biomedicals) with 10% fetal bovine serum (FBS) (Gibco) and 100 U/ml ampicillin–streptomycin with amphotericin B (Himedia). Primary mouse macrophages differentiated from circulated bone marrow–derived monocytes (MMa-bm) and macrophage medium (MaM) were procured from ScienCell Research laboratories (Carlsbad). The human macrophage cell line MD-CRL-9850 was purchased from American Type Culture Collection (Virginia), and the cells were grown in Iscove's modified Dulbecco's medium (Himedia) with 4 mM l-glutamine, 1.5 g/l sodium bicarbonate, 0.05 mM 2-mercaptoethanol, 0.1 mM hypoxanthine, and 0.016 mM thymidine supplemented with 10% FBS and 100 U/ml penicillin and streptomycin with amphotericin B. Mouse fibroblast NIH3T3 (a kind gift from Institute of Life Sciences) and mouse mammary epithelial cell line HC11 (generously gifted by Dr Sreenivasulu Kurukuti, Hyderabad Central University) were grown in DMEM and RPMI1640 medium, respectively, supplemented with 10% FBS. Mouse metastatic mammary tumor epithelial cell line 4T1 was purchased from American Type Culture Collection and maintained in RPMI1640 medium with 10% FBS. Caco2 is a human epithelial colorectal adenocarcinoma cell line (kindly gifted by Dr Kavitha Lole, National Institute of Virology), and it was cultured in minimum essential medium with 20% FBS; it differentiated into intestinal enterocytes when maintained after attaining confluency for a period of 15 to 21 days with periodical media change thrice a week. All cell lines were maintained at 37 °C with 5% CO_2_ in a humidified incubator. Cells were subcultured using 0.25% trypsin–EDTA solution (Himedia). LPS was purchased from Sigma–Aldrich. IFN-γ from Calbiochem, TNF-α from Peprotech, Ptx from MP Biomedicals, mouse recombinant GM-CSF protein (catalog no.: PMC2014) from Thermo Fisher Scientific, and STAT3 inhibitor S31-201 (catalog no.: sc-204304) from Santa Cruz Biotechnology were procured.

### Antibodies, plasmids, and siRNA

Anti-PELP1 antibody and anti-T7 antibody were purchased from Bethyl Laboratories, Inc. Vinculin was procured from Sigma–Aldrich and F4/80 (macrophage marker), anti-c-Rel, and β-actin antibodies from Santa Cruz Biotechnology. Anti-rabbit and antimouse horseradish peroxidase (HRP)-conjugated secondary antibodies were procured either from Santa Cruz Biotechnology or Rockland. Phospho p44/42 MAPK (catalog no.: 9106S), p44/42 MAPK (catalog no.: 9107S), Phospho STAT3 (catalog no.: 9134S), total STAT3 (catalog no.: 4904S), Phospho GSK3β (catalog no.: 5558S), and total GSK3β (catalog no.: 12456S) antibodies were purchased from Cell Signaling Technology. For IHC and immunofluorescence studies, anti-PELP1 antibody (IHC0013) was purchased from Bethyl Laboratories, and CD68 (AM416-5M) was purchased from Biogenex. Alexa Fluor 488, Alexa Fluor 546, and 4′,6-diamidino-2-phenylindole were procured from Life Technologies. Anti-GMCSF antibody was purchased from Abcam (catalog no.: Ab9741). The pGL3 Basic luciferase plasmid was purchased from Promega Corporation. Control siRNA and custom siRNA smart pool against PELP1 (L-041621-01-0005) and c-Rel (L-047122-00-0005 & EMU072641) were procured from GE Dharmacon and Sigma–Aldrich. Lentiviral particles (control [SHC001V] and PELP-1 targeting [TRCN0000159617]) for stable clone generation (MISSION shRNA) were purchased from Sigma–Aldrich.

### Western blotting

Protein extracts were prepared by cell lysis, utilizing radioimmunoprecipitation assay buffer (50 mM Tris–HCl [pH 7.4], 150 mM NaCl, 1% Nonidet P-40, 0.5% sodium deoxycholate, and 0.5% SDS) with protease inhibitor cocktail (Sigma) and phosphatase inhibitor (Roche). The protein concentration was estimated by using the DC protein estimation kit from Bio-Rad as described in the manufacturer's manual. Samples were subjected to SDS-PAGE and blotted onto nitrocellulose membrane. The membrane was blocked using 5% blocking buffer followed by specific primary antibody incubation overnight at 4 °C. Next day, after incubating the membrane with HRP-conjugated secondary antibody, the protein–antibody signals were visualized using an enhanced chemiluminescent assay kit (Bio-Rad) and processed using Quantity One Image system. The expression of housekeeping gene was further analyzed for normalizing the amount of protein loaded.

### Quantitative real-time PCR

Total RNA from tissues/cell lines was obtained by using TRIzol reagent (Thermo Fisher Scientific). Total RNA concentration of 0.5 to 1 μg was utilized for conversion into complementary DNA (cDNA) by using PrimeScript RT assay kit from Takara Bio, Inc. cDNA conversion reaction was carried out in a PCR cycler (SureCycler 8800; Agilent Technologies) at 37 °C for 15 min, and heat inactivation of the reverse transcriptase enzyme was at 85 °C for 5 s followed by storage at 4 °C. The cDNA obtained from RT reaction was diluted 2-fold to 10-fold, and 3 μl from the diluted cDNA was used to perform the qRT–PCR reaction by using Premix Ex Taq Perfect Real Time kit as mentioned in the manufacturer's protocol. Either gene-specific Taqman real-time PCR assay probes from Applied Biosystems (Mouse PELP1—Mm01245614_m1; Mouse Actin—Mm00607939) or primers for SYBR-green master mix were used for the real-time PCR. The RT–PCR was performed in an ABI 7500 real-time PCR system (Applied Biosystems). The relative gene expression was calculated using comparative ΔΔC_T_ approach after normalizing to the expression level of actin.

### Transfection and luciferase reporter assay

For promoter cloning, approximately ∼2 kb sequence upstream of the transcription initiation site was determined from sequence information given in National Center for Biotechnology Information/University of California Santa Cruz genome browser. The region is amplified and cloned into pGL3 basic luciferase vector (Promega). The genomic DNA from MCF7 and BALB/c mice were used as templates for the amplification of human and mouse PELP1 promoters for the selected regions, respectively. For human mutant promoter generation, potential transcription factor binding site was mutated using QuikChange II XL Site-6 Directed Mutagenesis assay kit from Agilent. The cells were transfected with appropriate amount of control plasmid or test plasmid DNA along with β-Gal plasmid DNA (0.5–5 μg) and incubated with FUGENE HD (Promega) (as given in the manufacturer's manual) in Opti-MEM at room temperature for 15 to 20 min. The cells were lysed in passive lysis buffer, and the lysate was used to assess the relative luciferase units after normalizing using β-Gal readings.

### ChIP

ChIP was carried out by using ChIP assay kit from Millipore (EMD Millipore) according to the manufacturer's protocol. In brief, the LPS-treated cells were fixed with 1% formaldehyde for 10 min at 37 °C. Immunoprecipitation was done with anti-IgG control and anti-c-Rel antibodies. The antibody-bound DNA was precipitated, PCR amplified using specific primers, and verified by sequencing.

### EMSA

EMSA was performed as described by the manufacturer (Pierce Biotechnology). Briefly, the biotin labeled, mutated, and unlabeled oligonucleotides containing the c-Rel–binding site were incubated with LPS-treated or untreated nuclear extracts from RAW 264.7 cells. The samples were resolved by native gel electrophoresis, and the DNA was transferred on to a nylon membrane followed by UV crosslinking. Finally, the bands were visualized by enhanced chemiluminescent detection system.

### siRNA transfection

Cells were transfected with scrambled control siRNA, and gene-specific siRNA of 20 μM stock concentration procured from Dharmacon On-TARGET plus siRNA's pool (Horizon Inspired Cell Solutions) using Lipofectamine RNAi Max (13778-075) (Thermo Fisher Scientific).

### RT^2^ profiler PCR array

Mouse IL6/STAT3 signaling pathway RT^2^ Profiler PCR Array was procured from Qiagen (SA Biosciences) and used to analyze the expression patterns of different key regulatory genes involved in inflammation. The microarray was performed as described by the manufacturer's protocol. RNA samples obtained from PELP1 siRNA and control siRNA-transfected cell lysates were used for the array. The fold change was calculated using comparative ΔΔC_T_ method by normalizing the data to an average of five housekeeping genes. RT^2^ Profiler PCR Data Analysis software was utilized to generate heat maps.

### Stable cell line generation and CM preparation

RAW 264.7 cells were transfected with pcDNA control and human PELP1 expression plasmids using Fugene HD reagent. The clones were selected using G418 antibiotic (MP Biomedicals) for 15 to 20 days followed by screening for T7-PELP1 by immunoprecipitation using T7-beads. The RAW 264.7 pcDNA and PELP1 clones were plated at 80% confluency in complete DMEM; the following day, media were replaced with DMEM without serum. The cells were allowed to grow for 48 h, then the cell supernatant was collected, concentrated, and stored in −80 °C for future use. 4T1 PELP1 KD clones and RAW 264.7 PELP1 KD clones were generated by transducing control and PELP1 shRNA lentiviral particles (MISSION shRNA; Sigma–Aldrich) with 4 μg/ml of polybrene (Sigma) as the transducing agent. The clones were selected using puromycin (MP Biomedicals) for 15 to 20 days followed by screening with Western blotting.

### ELISA

ELISA was performed to quantify GM-CSF expression in the CM. GM-CSF ELISA kit (catalog no.: ab100685) was purchased from Abcam Company and performed according to the instructions given in the manufacturer's manual. The experiments were run in duplicates, and the results were representatives of two independent experiments.

### GM-CSF depletion assay

Approximately 4 μg of anti-GM-CSF antibody was added to 2 ml of CM and incubated at 37 °C on rotation for 1 h. The cytokine–antibody complexes were removed by the addition of protein A/G beads (Santa Cruz Biotechnology) for 4 h at 4 °C on constant rotation. Samples were centrifuged, the supernatant was collected, filter sterilized, and used for subsequent experiments. Anti-IgG antibody was used as a negative control.

### Wound healing assay

NIH3T3 cells were plated at 90% seeding density in 35 mm culture plates and incubated at 37 °C in CO_2_ incubator. The following day, cells were treated with mitomycin C for 2 h followed by scratching using a p20 pipette tip. The cells were washed twice with PBS prior to the addition of concentrated CM. Cell migration was monitored, and images were captured at regular intervals by phase contrast microscope (Axio Vert.A1 system; Zeiss) connected to a camera.

### Transwell migration assay

Transwell cell migration assay apparatus contains a polycarbonate membrane insert of 8 μm pore size placed in a 24-well plate from Corning Inc. Cells with serum-free media were seeded into the inserts, and CM was added in the bottom well of the Boyden chamber apparatus. After 24 h of incubation, the migratory cells were stained and counted using bright field microscope. In GM-CSF–depletion experiment, the migration assay was executed with CytoSelect 24-well cell migration kit from Cell Bio labs, Inc following the manufacturer's instructions.

### Soft agar assay

Soft agar assay was achieved with CytoSelect 96-Well Cell Transformation Assay kit from Cell Bio labs, Inc as described in the manufacturer's protocol. The total number of transformed cells/well was calculated from the cells/milliliter in soft agar as given in the manual.

### IHC

All research studies on humans (samples or data) were performed in accordance with the principles stated in the Declaration of Helsinki. Prior to the start of the study, ethical approval was obtained from the local Institutional Review Board, SRIHER IEC (CSP/18/JAN/65/03). A progressive breast, cervix, and prostate cancer diseased spectra were procured from US Biomax, Inc, and clinical tissue samples for appendicitis, gastric, and colon cancer progression models were obtained with prior IEC approval. Formalin-fixed, paraffin-embedded tissue sections were mounted on precoated barrier slides, followed by deparaffinization, dehydration, and antigen retrieval. In the case of tissue microarray, the array slide was directly deparaffinized followed by antigen retrieval. The slides were blocked before incubating with IHC grade anti-PELP1 primary antibody (Bethyl Laboratories, Inc) at 4 °C overnight followed by HRP-conjugated anti-rabbit IgG antibody incubation. To this, peroxide/3,3′-diaminobenzidine substrate was added and incubated for 5 min followed by counterstaining with hematoxylin. In the case of double IHC (for double antigen labeling), protein blocking was followed by overnight incubation with the second primary antibody anti-CD68, Tris buffer washes, blocking, and incubation with AP polymer (ZytoMed). The reaction color was developed using Alk magenta substrate (Bio SB), counterstained, and mounted with DPX mountant. The intensity and percentage of cell staining was calculated and represented as Q score. Percentage of positive cells in double IHC ranged from 0 to 100, and the intensity of staining was graded 1+, 2+, and 3+ (weak, moderate, and high). Coexpression percentage was determined by percentage of cells expressing both PELP1 and CD68. The images were captured in Leica DM 2000 LED Light Microscope, and IHC images were captured using Microscope software platform Leica Application Suite at different magnifications.

### Double immunofluorescence

About 5 μm thick formalin-fixed and paraffin-embedded breast cancer tissue blocks were used for confocal-double immunofluorescence staining for PELP1 and CD68. Briefly, the slides were dewaxed in xylene followed by graded alcohol treatment. Antigen retrieval was carried out with heated high pH Tris–EDTA buffer method followed by hydrogen peroxide blocking and permeabilization using 0.4% Triton X-100. The slides were washed with 1× PBS and blocked. The slides were incubated with primary antibody cocktail for 1 h at room temperature. After washes, the slides were incubated with secondary antibody cocktail (Alexa Fluor 488 and 546). The slides were counterstained with 4′,6-diamidino-2-phenylindole and mounted using Vecta shield antifade mountant. Confocal images were captured by sequential scanning (Zeiss Stacking) under appropriate fluorescent filters on a Zeiss LSM 880 System and Zeiss Zen, version 3.2 software. Coexpression analysis (Pearson's correlation coefficient) was performed using ImageJ software.

### Animal study and mammary gland mounting

Animal experiments were carried out after obtaining clearance from the ethical review committee, IAEC/49/SRU/507/2016, and the international, national, and/or institutional guidelines were followed regarding the animal's welfare. Three doses of LPS (0.5 mg/kg body weight) and HC11 necrotic lysate (2.5 million HC11 cells/mice) were injected into the peritoneal cavity of Balb/c female mice for 3 consecutive days. This was followed by tumor induction by injecting 4T1 WT and 4T1 PELP1 KD clone cells (2.5 million cells/mice) into the mammary fat pads. The tumor growth was monitored at regular intervals. Weekly dose of LPS or HC11 necrotic lysate was given to keep the animal in the inflammation stimuli. After 3 to 4 weeks, the animals were euthanized, and the tumors were taken for pathological studies. Similarly, the experiment was done on athymic female mice. The mammary glands from the nude mice were mounted on super frost slides and stained with carmine dye to study the branching pattern of the gland as described by Tolg *et al*. (52).

### Statistical analysis

The data obtained from three independent experiments were expressed as SEM (mean ± SEM). The analysis was performed with Prism software, version 6 (GraphPad Software, Inc). The data significance between two or more groups was calculated using unpaired *t* test or one-way or two-way ANOVA. The *p* values were represented as statistically significant with ∗ (*p* ≤ 0.05), ∗∗ (*p* ≤ 0.01), ∗∗∗ (*p* ≤ 0.001), and ∗∗∗∗ (*p* < 0.0001). Statistical analyses for animal experiments and IHC studies were done with SPSS software package (IBM).

## Data availability

All representative data are contained within the article.

## Conflict of interest

The authors declare that they have no conflicts of interest with the contents of this article.
